# Supplementary material for: Ancient proteins provide evidence of dairy consumption in eastern Africa
Source: Nat Commun. 2021 Jan 27;12:632. doi: 10.1038/s41467-020-20682-3 (PMC7841170; doi:10.1038/s41467-020-20682-3)
Supplement: Supplementary file 1 — Supplementary Information [file 41467_2020_20682_MOESM1_ESM.pdf]

Supplementary Information

Ancient proteins provide evidence of dairy consumption in eastern Africa

Madeleine Bleasdale*, Kristine K. Richter, Anneke Janzen, Samantha Brown, Ashley Scott, Jana Zech, Shevan Wilkin, Ke Wang, Stephan Schiffels, Jocelyne Desideri, Marie Besse, Jacques Reinold, Mohamed Saad, Hiba Babiker, Robert C. Power, Emmanuel Ndiema, Christine Ogola, Fredrick K. Manthi, Muhammad Zahir, Michael Petraglia, Christian Trachsel, Paolo Nanni, Jonas Grossmann, Jessica Hendy, Alison Crowther, Patrick Roberts, Steven T. Goldstein, Nicole Boivin*

*Corresponding authors: Madeleine Bleasdale, Nicole Boivin.

Supplementary Note 1: Materials 2

Supplementary Note 2: Proteomic extraction methods 5

Supplementary Note 3: Oral Signature Screening Database (OSSD) 6

Supplementary Note 4: Stable carbon, nitrogen, and oxygen isotope analysis 11

Supplementary Figures:

Figures 1-8. Annotated spectra 16-23

Figure 9. $\delta^{13}C$ and $\delta^{18}O$ measurements for tooth enamel samples from Lukenya Hill 24

Figure 10. $\delta^{15}N$ and $\delta^{13}C$ measurements for bone collagen samples from Lukenya Hill… 25

Figure 11. $\delta^{13}C$ and $\delta^{18}O$ measurements for tooth enamel samples from Cole’s Burial 26

Figure 12. δ13C and δ18O measurements for tooth enamel samples from Molo Cave.............. 27

Figure 13. $\delta^{15}N$ and $\delta^{13}C$ measurements for bone collagen samples from Molo Cave 28

Figure 14. Radiocarbon date for Kadruka 1 SK68…. 29

Supplementary Tables:

Table 1. Summary of all dental calculus samples studied.. 30

Table 2. Summary of individuals with dairy proteins and radiocarbon dates……...................... 31

Table 3. Number of possible deamidation sites for milk proteins 32

Table 4. Summary of species information for peptide sequences 33

Table 5. Taxonomic information for species for casein proteins and BLG................................. 34

Table 6. Faunal identifications 35

References for Supplementary.. 36-39

Other Supplementary Materials for this manuscript include:

Supplementary Data 1-9 (provided separately as excel sheets).

Oral Signature Screening Database for Palaeoproteomic Analyses of Dental Calculus: Zenodo, [doi.org/10.5281/zenodo.3698271].

SP3 extraction protocol: protocols.io, [doi.org/10.17504/protocols.io.bfgrjjv6].

Supplementary Note 1: Materials

Kadruka 1 and Kadruka 21, Sudan

Kadruka is a Neolithic (~8000-5500 cal. BP), and Kerma period (~4450-3450 cal. BP) site located upstream of the 3rd Cataract of the Nile within the northern Dongola Reach. Fieldwork in the Kadruka District led by Jacques Reinold of the *Section française de la direction des antiquités du Soudan* (SFDAS) from 1985-2002 resulted in the discovery of 17 Neolithic Burial mounds. Of these, six mounds and over 700 individuals were excavated. At Kadruka 1, 124 individuals were excavated with approximately 70% (n = 96) from the Neolithic period and 30% (n = 46) form the Kerma period[1]. A total of 228 individuals were excavated at the cemetery site of Kadruka 21. For this study, a selection of skeletal remains (n = 10) were sampled: five each from Kadruka 1 (KDK 1) and Kadruka 21 (KDK 21), representing both the Neolithic and Kerma periods.

Preservation conditions at Kadruka have enabled the recovery of a large number of human remains in addition to a rich collection of material goods including cosmetic cases shaped from the canines of hippos, ivory handle tools, and painted vases[2]. The hot, dry climate of the site facilitated preservation of skin and hair, including hair on skeletal remains and preserved sheepskin[3,4]. At Kadruka 1, variations in burial arrangement and distribution of grave goods could indicate differences in the social stratigraphy[1]. One burial (KDK1/131), which was unavailable for sampling for this study, was located in the centre of the mound and contained a large quantity of grave goods including ivory bracelets, axe heads, ceramics, grindstones, and an Anthropoid sandstone figurine[1].

Collectively, the human remains from Kadruka were recovered from a funerary context, but zooarchaeological and botanical evidence has offered some important insights into the broad subsistence practices of the buried population. At Kadruka 1, sacks made from animal skin were discovered containing barley[2], and the remains of domestic sheep, goat, and cattle were reported[4]. Another potential line of evidence for the establishment of a dairy economy at the site is the presence of small perforated bowl (KDK1/120/3) from the Neolithic period[2] that bears similarities to Neolithic *"cheese strainers"* from Europe[5,6]. The Kadruka bowl was discovered filled with chaff and further analysis is needed to firmly establish its function.

Berber Meroitic Cemetery, Sudan

Berber Meroitic Cemetery is located on the east bank of the Nile River to the east of the centre of Berber City in northeastern Sudan. Archaeological excavations at the site started as a rescue project in 2009 in response to the construction of a plastics production factory. The subsequent discovery of a large, well-preserved Meroitic period cemetery instigated further excavations under the direction of Mahmoud Suliman Bashir of the *National Corporation for Antiquities and Museums* (NCAM) in Sudan. A number of substructures were uncovered during excavations including tombs and three mud brick pyramids. The total number of individuals buried at the site is still unknown as excavations are ongoing. Five individuals were analysed in this study but only one (BMC 38b) gave positive results for milk proteins. BMC 38 is the substructure of a mud brick structure, probably a pyramid, consisting of three courses with a funerary chapel located on the eastern side. Individual BMC 38b is an adult male buried in extended position east-west and was excavated close to another tomb directly dated to around 2160 cal. BP[7].

Lukenya Hill (GvJm 202), Kenya

Lukenya Hill is located in the Athi-Kapiti Plains east of the Central Rift Valley in southern Kenya. Lukenya Hill has several archaeological sites including rockshelters with Middle and Later Stone Age archaeology[8] and open-air Pastoral Neolithic (PN) sites[9,10]. For this study, dental calculus from five human teeth were analysed from the PN site of GvJm202. GvJm202 is a rockshelter containing the remains of at least six individuals consisting of five adults and one sub-adult[11,12]. Dental calculus was analysed from five teeth, including two discrete burials (Skeleton A and Skeleton C). The petrous portion of Skeleton C has been analysed for aDNA[13], and is dated to ~3635-3475 cal. BP[13]. Additionally, human bones from three individuals and seven teeth (including two teeth where milk proteins were identified in associated dental calculus) were analysed for stable isotope analysis. Bone collagen from three *Bos* specimens (identified using morphology and ZooMS: see methods) from Lukenya Hill was also analysed for stable carbon ($\delta^{13}C$) and nitrogen ($\delta^{15}N$) isotope composition: two *Bos* specimens are from GvJm202 and one is from the neighbouring PN site of GvJm184. GvJm184 is dated to ~2715-1735 cal. BP[10,14] and has produced Savanna Pastoral Neolithic (SPN) pottery and the remains of domesticated cattle, sheep, and goat[14].

Cole's Burial (GrJj5a), Kenya

Cole's Burial site is located on the eastern side of Lake Elmenteita in central Kenya[15]. Charles Nelson and Stanley Ambrose documented the site in 1976 when fragmentary

human remains were observed in the cliffs above the lake. A minimum of three individuals were excavated. A tibia from one individual (CB1) was previously directly radiocarbon dated to 2750-2015 cal. BP on apatite (2355 ± 150 BP; GX4714-A) and 2854-2185 BP on gelatin (2500 ± 130 BP; GX4714-G)[15]. Genetic analysis and radiocarbon dating (3350-3180 cal. BP; 3070 ± 20 BP; PSUAMS4723) was carried out previously on CB1.01[16]. Bone collagen from all three individuals was analysed previously using stable isotopes of carbon and nitrogen[14]. For this study, dental calculus was sampled for proteomic analysis from two burials (CB1.01 hereafter Individual 1, and Individual 2) and from a loose incisor not associated directly with any of the three burials. To further investigate dietary intake, tooth enamel from Individual 1 and the isolated tooth were was analysed using stable carbon ($\delta^{13}C$) and oxygen ($\delta^{18}O$) isotope analysis. Additionally, enamel was sampled from a rodent and mole rat (*Tachyoryctes*).

Molo Cave (GoJi 3), Kenya

Molo Cave is located approximately 50 km west of Lake Nakuru in the Central Rift Valley of southern Kenya. The remains of three individuals were salvaged by Mary D. Leakey and are presently curated by the National Museums of Kenya. The individuals are believed to be associated with the Pastoral Neolithic period[11]. For this study, dental calculus was sampled from one discrete burial (Skeleton 1) and one loose tooth. Skeleton 1 has been analysed previously for aDNA[13] and directly radiocarbon dated to 1415-1320 cal. BP (OxA-37, 359) (Supplementary Table 2)[13] supporting the likely Pastoral Neolithic attribution. Tooth enamel from this individual was analysed as part of this study using stable carbon ($\delta^{13}C$) and oxygen ($\delta^{18}O$) isotope analyses along with enamel and bone collagen from two other individuals and a range of wild and domesticated fauna (*Bos, Cephalophus*, *Capra hircus*, *Dendrohyrax,* and *Heterohyrax)* (Supplementary Information Section 4, Supplementary Figs.12-13).

Supplementary Note 2: Proteomic Extraction Methods

Two proteomic extraction methods were used in this study: FASP and SP3. Modified FASP (Filter-Aided Sample Preparation)[17] protocols have successfully been applied in several ancient dental calculus studies[18-20], including the first study to report milk peptides[21]. However, filter-based protocols are less suited to small amounts of starting material[22]. Recently, Single-pot, solid-phase-enhance sample preparation (SP3) has been developed to overcome sample size limitations of FASP[23,24]. However, this method is based on protein precipitation and aggregation[25], which could be limited or absent in cases where proteins are heavily fragmented. For a full description of SP3, see Methods and protocol published on protocols.io [doi.org/10.17504/protocols.io.bfgrjjv6].

In this study, dental calculus samples from Kadruka (n = 10) were extracted using both methods. For other sites, sample were extracted only with SP3 because start weights were too low for FASP. While this is the first study using SP3 on archaeological dental calculus and differences were observed between the methods, statistical comparisons were not conducted due to the small number of samples prepared with both methods.

Supplementary Note 3: Oral Signature Screening Database (OSSD) for the Palaeoproteomic Analysis of Dental Calculus

Full methods, database, and results of pilot test available at open-access repository Zenodo [doi.org/10.5281/zenodo.3698271].

As the proteomic analysis of dietary peptides retrieved from ancient dental calculus becomes more common[19-21,26], new methods of authentication need to be considered. Here we present an Oral Signature Screening Database (hereafter OSSD) developed as a screening tool for ancient dental calculus.

A major challenge in palaeoproteomics of dental calculus is to show that the dietary proteins reported are endogenous (i.e. they became entrapped in the calculus during formation) as opposed to modern contamination. One method to investigate whether such proteins are truly "ancient" is through the estimation of deamidation rates of glutamate and asparagine[27,28]. This method has been used to assess proteins identified from ancient dental calculus samples from the United Kingdom and Mongolia[19,20,29]. However, it is more challenging to apply similar methods to assess poorly preserved samples due to a number of limitations. Firstly, it requires a large number of endogenous peptides in order to have adequate deamidation sites for statistical models. Secondly, it cannot authenticate individual peptides, only the entire identified sample or a sufficiently large subset of an identified sample. Finally, due to variations in deamidation rates between different peptides and different sites within a peptide[30], it is best suited to samples that have a high coverage of a small number of proteins.

The dental calculus samples in this study had a lower total number of proteins recovered when compared to published results for other geographical and archaeological contexts[19,20,26]. Consequently, there is a low percentage of endogenous peptides overall meaning a small number of deamidation sites. We therefore considered an alternative way to screen calculus samples in order to quickly identify potentially problematic samples as well as those which are more likely to yield proteins of endogenous origin. As reported in the literature, well-preserved dental calculus samples include human oral proteins and oral microbiome proteins[31]. However, this “oral signature” is not routinely reported in a standardised format in palaeoproteomic studies. In part, this is because the oral microbiome is diverse with reported differences between the oral microbiomes of modern plaque and ancient calculus[32]. Therefore, we selected a restricted list of the most abundant oral signature proteins and microbial proteomes seen in ancient dental calculus. This enabled us to produce a screening database requiring minimal computational time.

The OSSD includes proteomes from a subset of the most common oral microbes, human inflammatory response proteins commonly found in archaeological samples and contaminants introduced during laboratory preparation (trypsin) or handling (keratins). The protein list for the database was created by finding commonalities amongst published datasets for dental calculus[18,21,26], as well as unpublished results generated by the Palaeoproteomics Lab Group in Jena (MPI-SHH).The full list of proteins in the database are available on Zenodo [doi.org/10.5281/zenodo.3698271]. Proteins were divided into four categories: lab contaminants, common contaminants, oral microbiome, and immune response. Common lab contaminants include trypsin, the enzyme used during the extraction process, and serum albumin which is often a contaminant in modern proteomics facilities. The common contaminants list includes collagens and keratins which are introduced through sample handling and proteins associated with the burial environment.

The primary aim of the OSSD is to provide a quick screening method to authenticate the oral signature in archaeological dental calculus samples. While we acknowledge oral biomes can contain numerous bacterial species and be highly variable, it is not the purpose of the OSSD to fully capture this diversity. Therefore, we only selected a subset of common oral bacteria identified in ancient dental calculus samples. This included the three members of the “red complex” (*Porphyromonas gingivalis, Treponema denticola*, and *Tannerella forsythia*) which are associated with periodontal disease[33,34] and other commonly identified microbes (*Actinomyces naeslundii, Treponema maltophilum, Streptococcus mutans, Streptococcus gordonii*, and *Methanobrevibacter oralis*). In order to ensure a short run time (<30 mins) for the OSSD when used with common MS/MS data analysis tools, we selected 11 bacteria proteomes in total. We recognise the list of oral bacteria is not extensive and that the OSSD is not a substitute for the comprehensive oral database eHOMD (expanded Human Oral Microbiome Database) which contains over 700 microbial species[35]. The OSSD is a screening tool and we would therefore recommend the use of other databases, such as eHOMD, for in-depth assessment of bacterial proteomes. In addition to oral bacteria proteomes, we also included two human proteins (lysozyme C and lactotransferrin) and 10 human immune proteins (immunoglobulin kappa constant, neutrophil elastase, cathepsin G, antithrombin-III, alpha-1-antitrypsin, myeloperoxidase, neutrophil defensin, S100-A9, S100-A8, and complement C3) commonly identified in palaeoproteoimc calculus samples[31].

The OSSD was tested on a number of published dental calculus samples and associated blanks: 11 samples from PXD009603[26], 15 samples from PXD012893[19], and 14 samples from PXD008217[18]. In addition, we tested it against published results of archaeological and modern bones and sediments: 16 from PXD014657[36] and 12 from [37] MassIVE

MSV000083687 [doi:10.25345/C5G04C], as well two internal bone extractions (unpublished). Samples were extracted with different methods (gelatinisation, GASP, FASP, SP3) in different laboratories, and are from modern and archaeological contexts from across the world.

The database was tested on Byonic Protein Metrics Inc.[38] with the following settings: non-specific digestion; a precursor mass tolerance of 5ppm; a fragment mass tolerance of 0.05Da, carbamidomethyl of cysteine as a fixed modification; variable modifications (2 common, 1 rare) as deamidation of asparagine and glutamate (2 common); oxidation of lysine and methionine (2 common); phosphorylation of serine and threonine (1 common); glutamate or glutamic acid to pryo-glutamate (1 rare); and acetyl at the n-terminus (1 rare). Proteins were manually assigned to each of the four categories and totals calculated. Proteins were considered authentic if they had at least four peptides assigned and had a log probability of greater than one, or greater than the highest scoring decoy, whichever was higher.

Proteins were considered authentic if they had at least four peptide spectral matches (PSMs) assigned and had a log probability of greater than one, or greater than the highest scoring decoy, whichever was higher. In the blank samples, there were no oral signature proteins and the total number of proteins was between 0 and 25. Over all of the samples, the average number of contaminant proteins was 5.5. Therefore, in order to pass OSSD cut-off (demonstrating that there was a “real” oral signature), samples required at least 10 total proteins with 45% of proteins assigned to the oral microbiome or immune response protein subcategories. In addition, samples that passed this threshold were assigned an OSSD score consisting of three levels, low, medium and high quality (Supplementary Data 3). The levels were as follows: low (10-19 proteins in total with <75% assigned to oral microbiome or immune response protein categories); medium (20-49 proteins in total with >75% assigned to oral microbiome or immune response protein categories or 50+ proteins in total with 75-90% assigned to oral microbiome or immune response protein categories); and high (50 or more total proteins with >90% assigned to oral microbiome or immune response protein categories).

The results of the pilot test were concordant with expectations; all bones and blank samples failed to meet the OSSD threshold (for full results see Zenodo [doi.org/10.5281/zenodo.3698271]). Thirty-one out of thirty-two calculus samples passed our OSSD threshold, including all calculus samples that were reported in the published literature to have milk peptides.

At this time, the OSSD is not comprehensive and requires further development. As more ancient dental calculus results are published, the database will be tested, refined, and new versions will be made available. Additionally, more testing is needed to identify cut-off values for authenticity of the oral signature for different methods and regions of the world. Finally, this method does not overcome the problem of needing to authenticate individual peptides. Samples which have an endogenous oral signature could still be contaminated with peptides from modern food sources. Additionally, using the whole proteome of oral microbes likely allows for overlap between proteins found in both oral microbes and soil microbes. Nevertheless, for this study, the OSSD provided a quick method to screen and quantify any possible oral signature in the calculus samples, enabling the elimination of the most poorly preserved samples.

Additional Information on Milk Peptide Identifications

In previous studies of archaeological dental calculus, positive identifications of dietary proteins in an individual have required at least two unique peptide sequences[18–20,26]. However, these studies are largely based on well-preserved samples from Europe or Asia. Our study is the first to analyse ancient dental calculus from Africa with samples exhibiting a far lower total number of preserved proteins than those from other geographical contexts; we therefore modified this criterion in our study.

Across previously published calculus studies, the milk peptide beginning at position 143 (TPEVDDEALEK) is most frequently observed (both in ancient and modern samples) while other peptides are observed at a lower frequency[26,39]. As our samples had a lower overall level of preservation, we predicted to see a smaller subset of the possible recoverable peptides[40,41]. We therefore relaxed previously used parameters for a positive indication by allowing one unique sequence if, and only if, that sequence was the peptide starting at position 143. For all other protein identifications, two unique sequences were still required. We considered individuals to have authentic dairy proteins if the individual passed the OSSD, had at least four milk peptides (two unique sequences or all four PSMs starting at position 143) at least two of which were identified in both Mascot and Byonic.

In some cases, we are able to confirm the likely presence of cow, sheep, and goat's milk (Supplementary Table 4, Supplementary Table 5, Supplementary Data 6). Species-specific peptides were recovered from one sample. Other identifications could only be associated with broader taxonomic categories. BLG is the most frequently identified milk protein in archaeological dental calculus, but many of the commonly recovered peptides such as TPEVDDEALEK[18-20] are shared among a variety of taxa. Additionally, post-translational

modifications such as deamidation of asparagine can occur through normal sample processing, which make it difficult to determine species with these peptides[26]. However, when these peptides are present with sufficient b and y-ion series coverage, species identifications can be made confidently.

Supplementary Note 4: Stable carbon, nitrogen, and oxygen isotope analysis

Stable carbon and nitrogen isotope analysis of human and faunal bone collagen

The stable isotope ($\delta^{13}C$ and $\delta^{15}N$) analysis of bulk bone collagen from humans and animals from the archaeological record has been widely applied to investigate dietary variation in the past. In terrestrial ecosystems there is an isotopic distinction between the two major photosynthetic pathways, C_3 and C_4, which differ in their net discrimination against ^{13}C during the fixation of CO_2. C_3 plants have highly negative and variable $\delta^{13}C$ values (ranging from around -35‰ to -19‰). Plants using the C_4 photosynthetic pathway have higher values ranging from -8‰ to -13‰[42–45]. The differences between these two groups of plants is passed into the tissues of their consumers with the $\delta^{13}C$ of bone collagen being around 5‰ more positive than the diet[46,47].

The $\delta^{15}N$ of bone collagen reflects differences relating to the trophic level of consumers, increasing by approximately 3-5‰ with each trophic level[48] and therefore $\delta^{15}N$ measurements can be also used to separate consumers feeding exclusively on marine sources compared to terrestrial sources[49]. Furthermore, there is also a slight enrichment of 0–2‰ in $\delta^{13}C$ as trophic levels increase[48]. Determining freshwater fish consumption is more challenging due to middling $\delta^{15}N$ and $\delta^{13}C$ values. Generally, the consumption of freshwater fish will result in higher bone collagen $\delta^{15}N$ values, but the complex cycling of carbon in freshwater systems means there can be significant variation in $\delta^{13}C$ [50].

Stable carbon and oxygen isotope analysis of human and faunal tooth enamel

While $\delta^{13}C$ of bone collagen largely reflects the protein portion of an individual's diet, the $\delta^{13}C$ of carbonate in the bioapatite of tooth enamel is more representative of overall dietary intake including carbohydrates, proteins, and lipids, meaning that low protein foodstuffs such as crops may be more represented in human bioapatite than collagen[51]. Additionally, it is possible to measure the $\delta^{18}O$ of enamel that is reflective of ingested water (as water or food sources), as the water consumed is closely related to the isotopic composition of local precipitation, $\delta^{18}O$ measurements can therefore provide information about the environment and mobility[52–54].

Stable isotope approaches to investigate pastoralism in Africa

Stable isotopes have been used to explore the emergence and development of pastoral lifeways in Africa through the analysis of archaeological remains. The isotopic analysis

($^{87}Sr/^{86}Sr$, $\delta^{13}C$, $\delta^{18}O$) of human and faunal tooth enamel has been used to investigate pastoral mobility in the central Sahara[55], South Africa[56,57], and Kenya[58], and has revealed a more complex picture for the emergence of early herding in relation to ecological change[59]. Bulk bone collagen isotope analyses have been used to investigate the emergence of cattle-based pastoralism in southern Africa[60] and tease apart different subsistence strategies by analysing prehistoric and historic communities in Africa practicing herding, fishing, and farming[14,61]. Herders, who rely heavily on animals and animal products, generally have higher $\delta^{15}N$ than communities relying heavily on plant products. Furthermore, higher $\delta^{13}C$ in human collagen could reflect a reliance on grazers consuming local C_4 grasses[14,61].

Here we analysed humans from the three sites in Kenya that produced proteomic evidence for milk consumption: Lukenya Hill, Cole's Burial, and Molo Cave. While dietary proteins from dental calculus provide "snapshots" of dietary intake, isotope measurements of different tissues can be used to look at reliance on different food sources. In addition, bone collagen and tooth enamel were sampled from a range of local fauna to provide baselines against which human dietary signals could be examined. As some faunal remains were highly fragmented, we used Zooarchaeology by Mass Spectrometry (ZooMS) to improve taxonomic identifications. Of particular interest in this study were domestic dairy animals such as cattle, goats, and sheep, as well as any available wild fauna as a reference. $\delta^{13}C$ isotope analyses can be used to distinguish between different domesticates based on feeding behaviours. For example, modern sheep at low elevations in eastern Africa display higher $\delta^{13}C$, while goats have more variable values due to consuming mixed C_3/C_4 diet[62]. Furthermore, isotopic studies of faunal remains from sites across Kenya suggest that cattle (C_4-grazers) display minimal variability in diet, in contrast to other grazers (sheep and goats) which could have greater diversity[58,63]. Integrated approaches (such as those combining ZooMS and isotopes) appear most effective in archaeological contexts for confidently distinguishing species[64].

In this study, human $\delta^{13}C$ values were compared to associated fauna. Bone collagen $\delta^{13}C$ largely reflects protein intake because dietary amino acids are preferentially used for the construction of collagen[51,65]. For individuals from Pastoral Neolithic sites in Kenya, we would therefore expect alignment between the $\delta^{13}C$ of domestic livestock (sheep, goats, and cattle) and humans. Furthermore, we would anticipate that human $\delta^{15}N$ would be indicative of the consumption of terrestrial protein sources due to the dominance of animal products in the diet of pastoralists.

Results

All isotope results are summarised in Supplementary Data 8 and Supplementary Data 9. Identifications of the faunal remains are presented in Supplementary Table 6 and Supplementary Data 7. Isotope samples were subjected to a series of quality controls, these included a C/N ratio of 2.9-3.6, %C of ca.15-48%, and %N of ca. 5-17%[42,66,67]**.** Thirteen out of fourteen bone samples passed quality checks and were carried forward for analysis.

Kadruka 1

A bulk hair sample from Kadruka 1 Skeleton 68 was sampled for dating at Centre for Isotope Research (CIO) Groningen. Additionally, a subsample was analysed using stable carbon and nitrogen isotopes to explore dietary intake. The results ($\delta^{13}C$ -17.0‰, $\delta^{15}N$ 12.0‰) broadly indicate the consumption of C_3-based dietary sources (animals feeding on C_3 resources or C_3 plants).

Lukenya Hill (GvJm202 and GvJm184)

Two individuals from Lukenya Hill (GvJm202) had BLG derived from Bovinae/*Ovis*. For these individuals it was also possible to analyse tooth enamel (Supplementary Fig.9, Supplementary Data 8). Their $\delta^{13}C$ enamel values of -3.7‰ and -0.3‰ are higher than the $\delta^{13}C$ value of -6.7‰ from the Thomson's gazelle (*Eudorcas thomsonii*), a mixed feeder, and suggest an almost entirely C_4-based diet. Bone collagen from an additional three individuals from the site that did not yield BLG proteins display $\delta^{13}C$ values of -6.6‰ to -5.7‰, again supporting a diet of largely C_4 protein sources (Supplementary Fig.10). These are consistent with bulk collagen results from the same site published in[14]. The human $\delta^{13}C$ values were similar to those obtained from animal remains recovered from the site (-6.5‰ to -5.8‰) which were identified as *Bos* sp. using ZooMS (Zooarchaeology by Mass Spectrometry, see Methods). For humans, the $\delta^{15}N$ for is 12.7‰ compared to a mean $\delta^{15}N$ value of 8.0‰ for the *Bos* specimens from Lukenya Hill. *Bos* bone collagen $\delta^{13}C$ values are consistent with the consumption of C_4 grasses (Supplementary Fig.10). While interpretations are somewhat limited due to the absence of any collagen data from wild fauna from Lukenya Hill, when all human and faunal $\delta^{13}C$ and $\delta^{15}N$ values are considered together they suggest a reliance on grazing animal products rather than cereal crops.

Cole's Burial (GrJj 5a)

Bone collagen from this individual produced a $\delta^{13}C$ value of -4.5‰[16] indicating a diet based on C_4 food protein sources. For this study, the tooth enamel of Individual 1 was analysed because $\delta^{13}C$ from enamel is more reflective of the whole diet[65]. The individual produced a $\delta^{13}C$ value of -1.2‰, confirming that this individual ate a primarily C_4-based diet. Tooth enamel was also analysed from an isolated tooth and gave a $\delta^{13}C$ -3.4‰ (Supplementary Fig.11) indicative of a diet with a high proportion of C_4 sources. In addition to the humans, tooth enamel was sampled from two rodents (including one mole rat (*Tachyoryctes* sp.)), these were the only fauna available for sampling. The rodents produced $\delta^{13}C$ measurements of -1.2‰ and -4.8‰ that broadly correspond to the values of the humans and, again, suggest the consumption of largely C_4 sources. Bone collagen from two individuals from Cole's Burial was analysed using stable isotopes carbon and nitrogen and reported in[14] and collagen from CB1.01 (in this study Individual 1) produced a $\delta^{13}C$ value of -4.5‰[16]. All three samples produced $\delta^{13}C$ between -4.0‰ and -5.0‰ indicating a high reliance on C_4 sources. Although in this case, distinguishing reliance on animals is challenging given the lack of faunal data.

Molo Cave (GoJi 3)

Tooth enamel samples were taken from three human individuals from Molo Cave, including Skeleton 1 that had BLG peptides in their calculus (this study) and is radiocarbon dated to 1415–1320 cal BP[13]. To contextualise the human isotopic results, a range of fauna were sampled from the site representing different feeding niches (arboreal browsers and grazers), which can be separated out according to their $\delta^{13}C$ enamel values. The *Dendrohyrax* and *Heterohyrax*, with $\delta^{13}C$ measurements of -18.3‰ and -14.1‰ respectively, reflect diets comprised largely of C_3 plants. The duiker (*Cephalophus*) $\delta^{13}C$ value (-12.4‰) is consistent with the consumption of largely C_3 sources (Supplementary Fig.12). In contrast, the two *Capra hircus* and one *Bos* specimens ($\delta^{13}C$ ranging from -1.7‰ to 0.5‰) are animals grazing on C_4 grasses. When comparing humans to the fauna, all individuals' (including Skeleton 1) $\delta^{13}C$ values are close to those of the domestic species (*Capra* and *Bos*). To further explore the degree to which individuals buried at Molo Cave relied on animal products (meat and dairy), bone collagen from two individuals was analysed along with associated fauna (Supplementary Fig.13). The humans have similar $\delta^{15}N$ collagen values (11.4‰ and 11.8‰). When considered against the $\delta^{15}N$ of the livestock (*Ovis*: 8.3‰, *Bos* average: 6.6‰), the isotope results suggest that the humans consumed a diet of terrestrial protein. The fact that the humans' isotope values (both $\delta^{15}N$ and $\delta^{13}C$) appear to align with

the domestic fauna supports the assertion that these individuals relied on animals or animal products to a large degree.

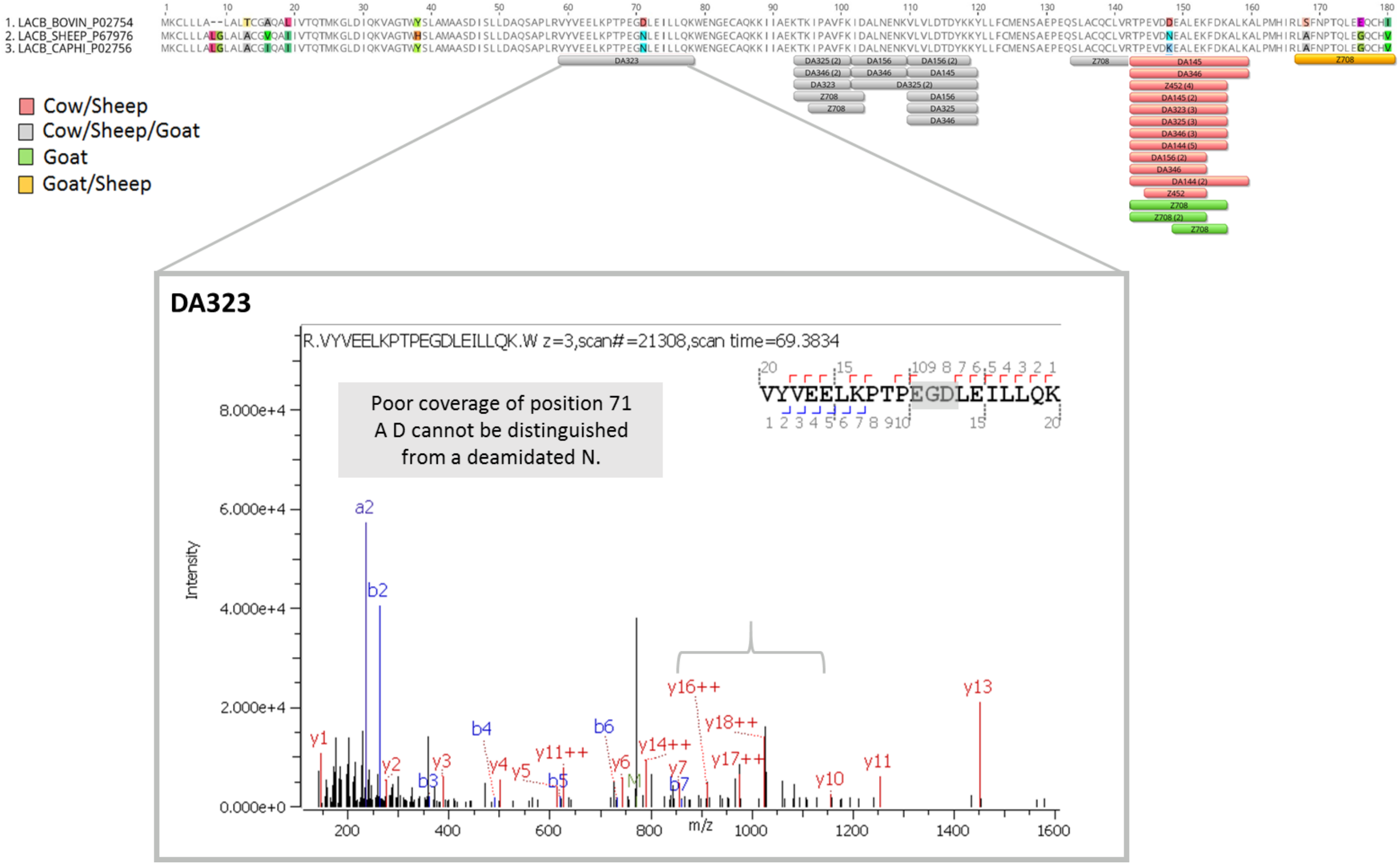

Supplementary Figure 1: Above: Alignment map for all BLG (LACB) peptides by individual. Species-specific information is indicated by different colours. Below: Annotated spectra for sample DA323. Brackets show B and Y ions difference corresponding to highlighted amino acids.

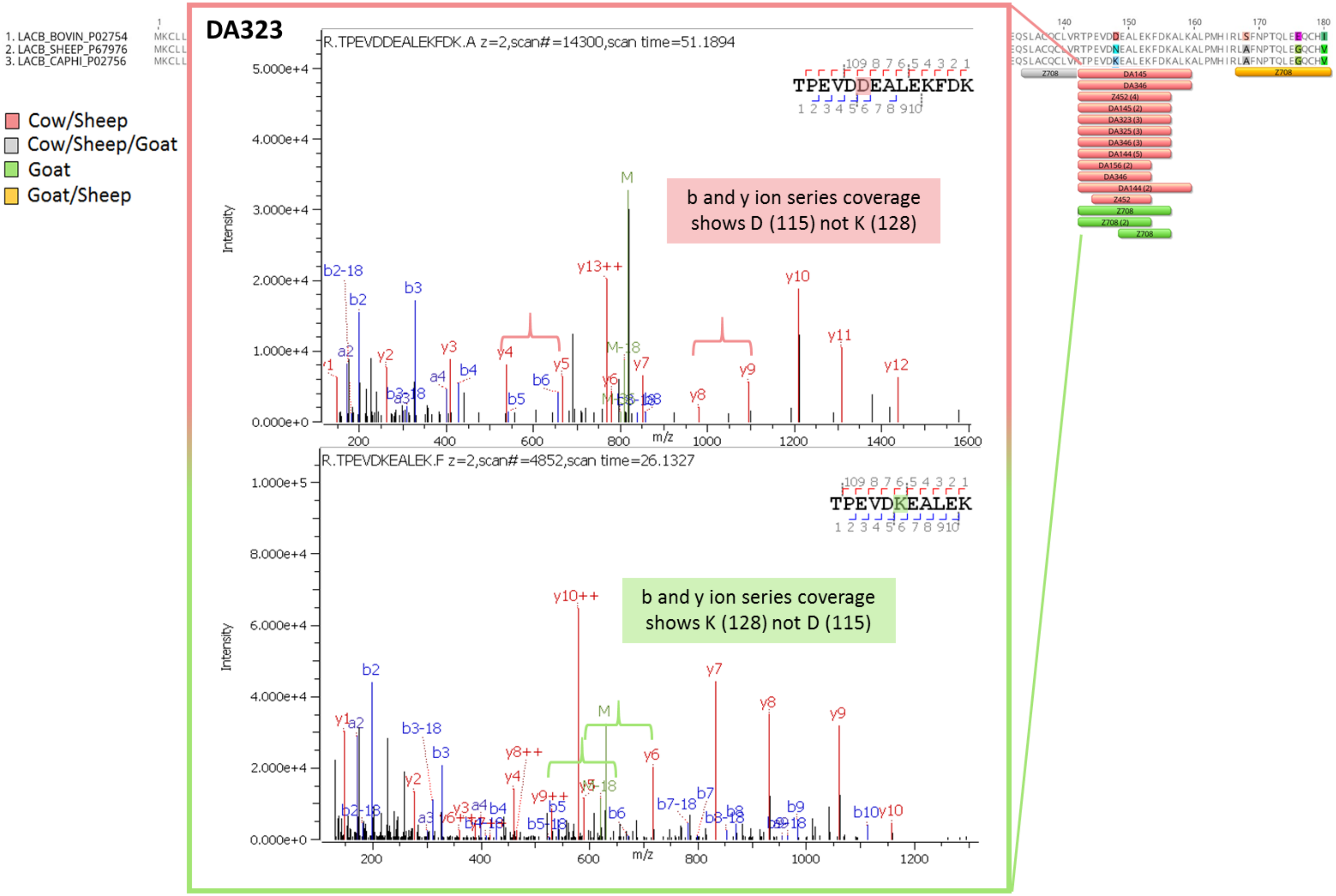

Supplementary Figure 2: Above: Alignment map for all BLG (LACB) peptides by individual. Species-specific information is indicated by different colours. Below: Annotated spectra for sample DA323. Brackets show B and Y ions difference corresponding to highlighted amino acids.

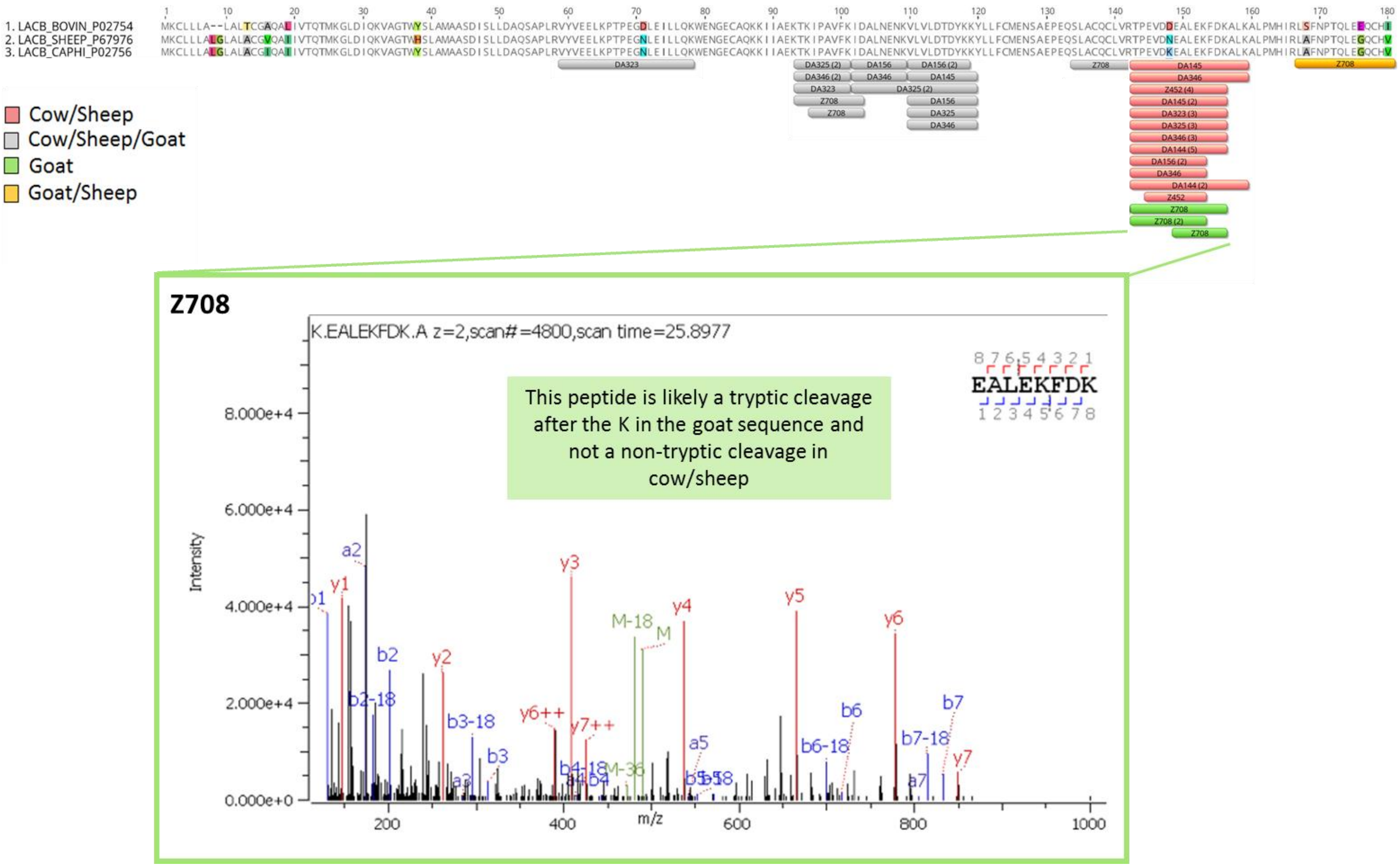

Supplementary Figure 3: Above: Alignment map for all BLG (LACB) peptides by individual. Species-specific information is indicated by different colours. Below: Annotated spectra for sample Z708.

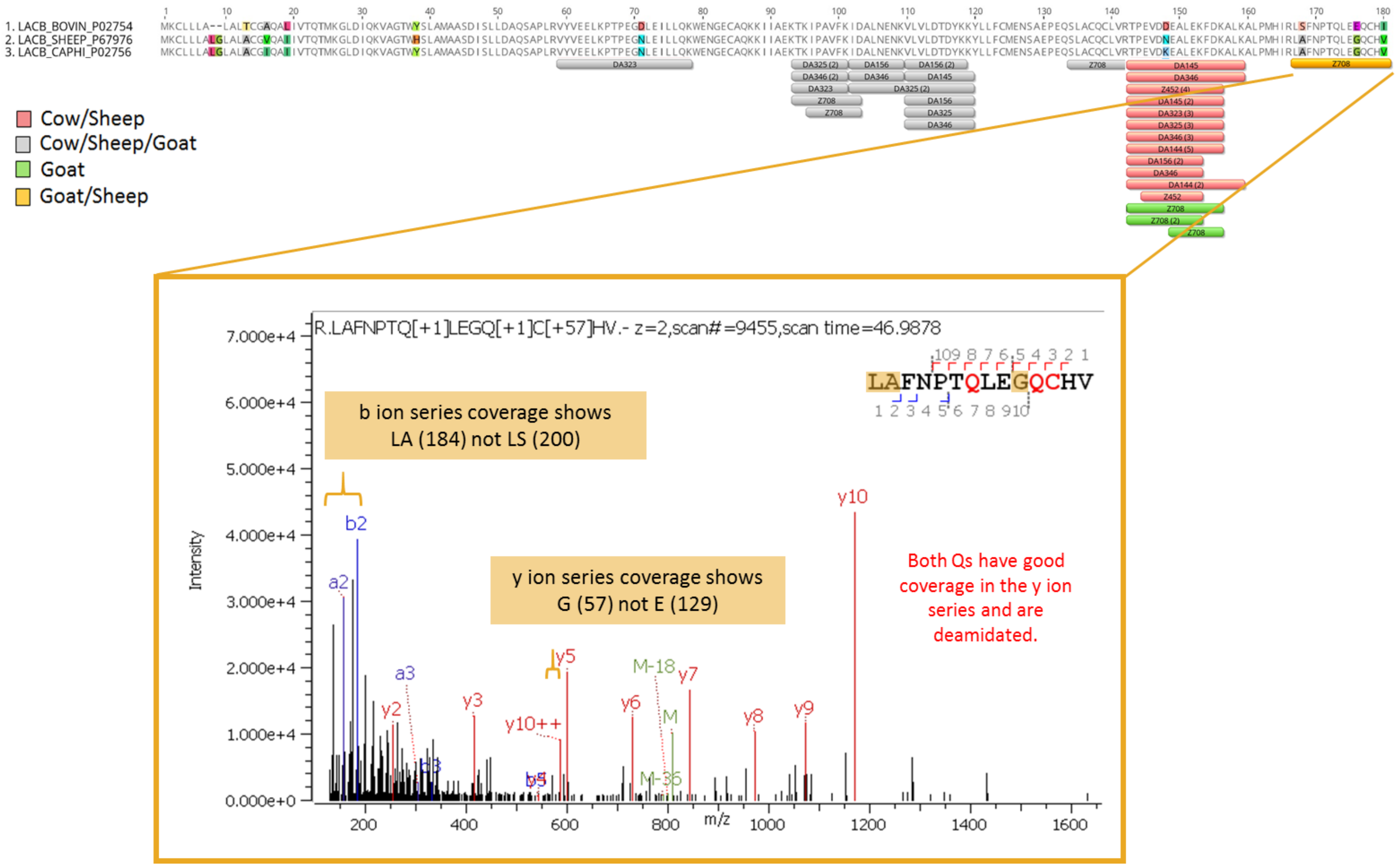

Supplementary Figure 4: Above: Alignment map for all BLG (LACB) peptides by individual. Species-specific information is indicated by different colours. Below: Annotated spectra for sample Z708. Brackets show B and Y ions difference corresponding to highlighted amino acids.

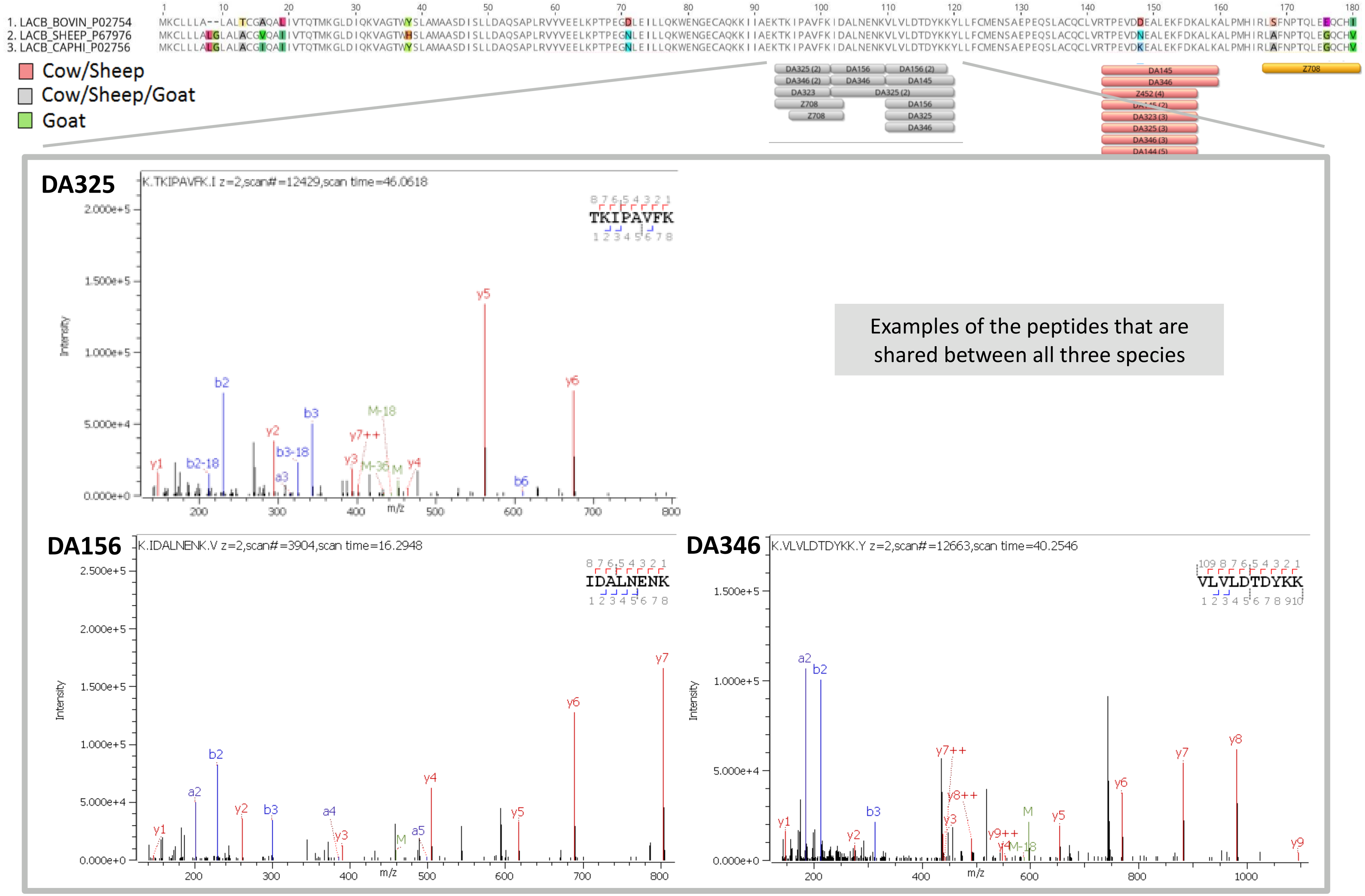

Supplementary Figure 5: Above: Alignment map for all BLG (LACB) peptides by individual. Species-specific information is indicated by different colours. Below: Annotated spectra for samples DA325, DA156 and DA346.

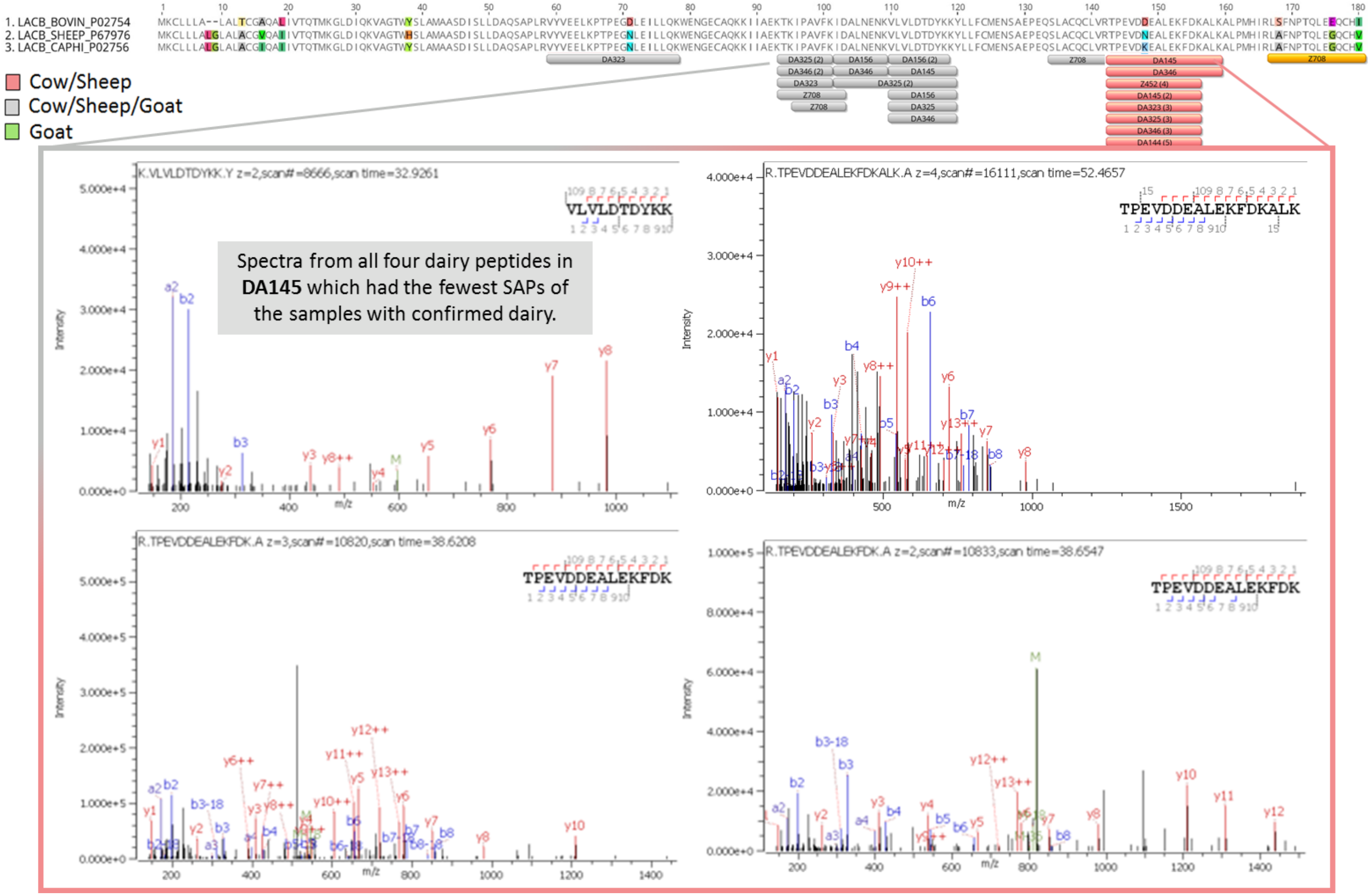

Supplementary Figure 6: Above: Alignment map for all BLG (LACB) peptides by individual. Species-specific information is indicated by different colours. Below: annotated spectra from all four dairy peptides in sample DA145.

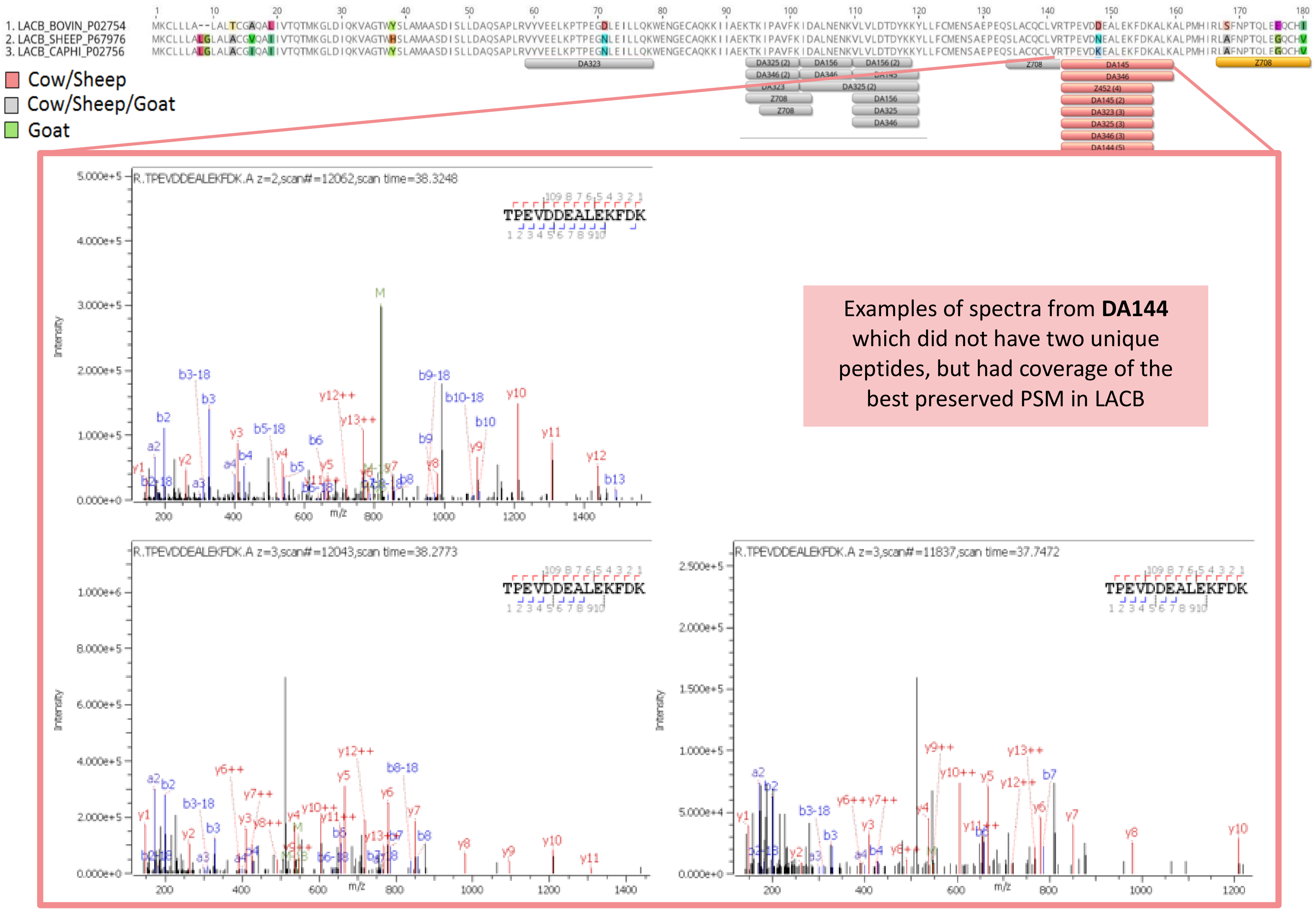

Supplementary Figure 7: Above: Alignment map for all BLG (LACB) peptides by individual. Species-specific information is indicated by different colours. Below: annotated spectra for sample DA144.

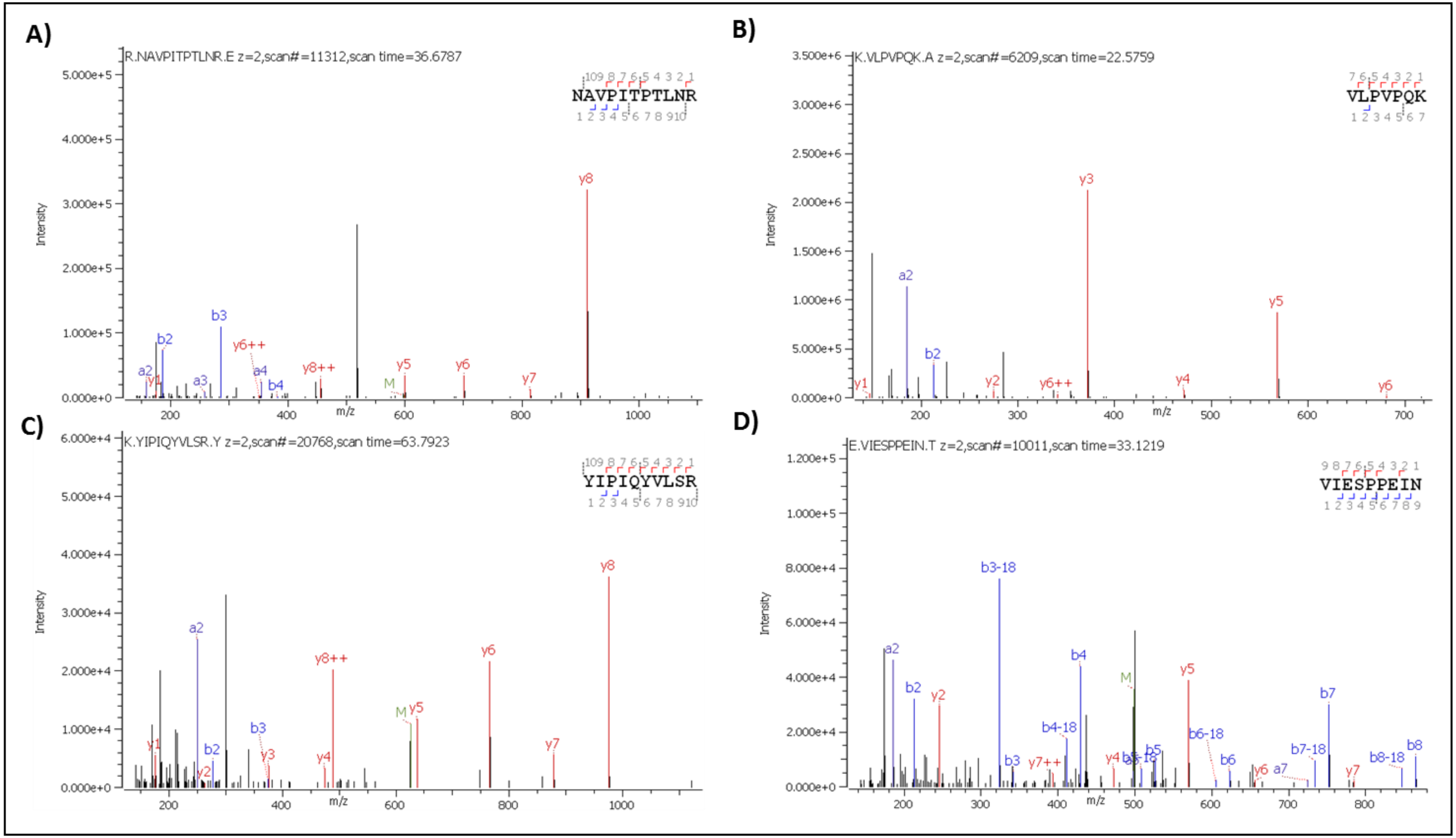

Supplementary Figure 8: Examples of spectra for casein peptides from sample DA156: A) Alpha-S2-casein (CASA2); B) beta-casein (CASB); C) kappa-Casein (CASK); D) kappa-Casein (CASK) identified only with non-tryptic search with Byonic.

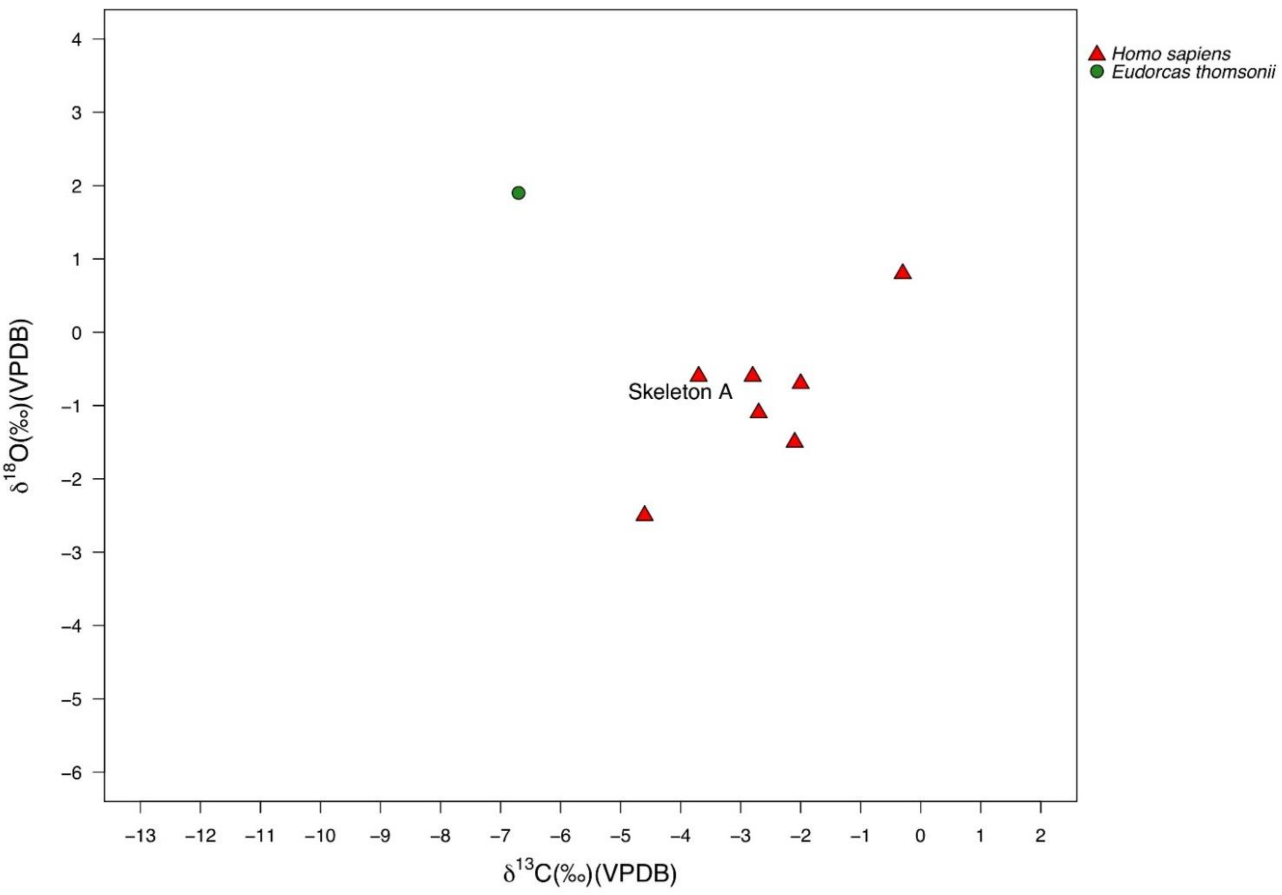

Supplementary Figure 9: $\delta^{13}C$ and $\delta^{18}O$ measurements for tooth enamel samples from humans and a Thomson's gazelle (*Eudorcas thomsonii*) from Lukenya Hill. Skeleton A produced proteomic evidence of milk consumption.

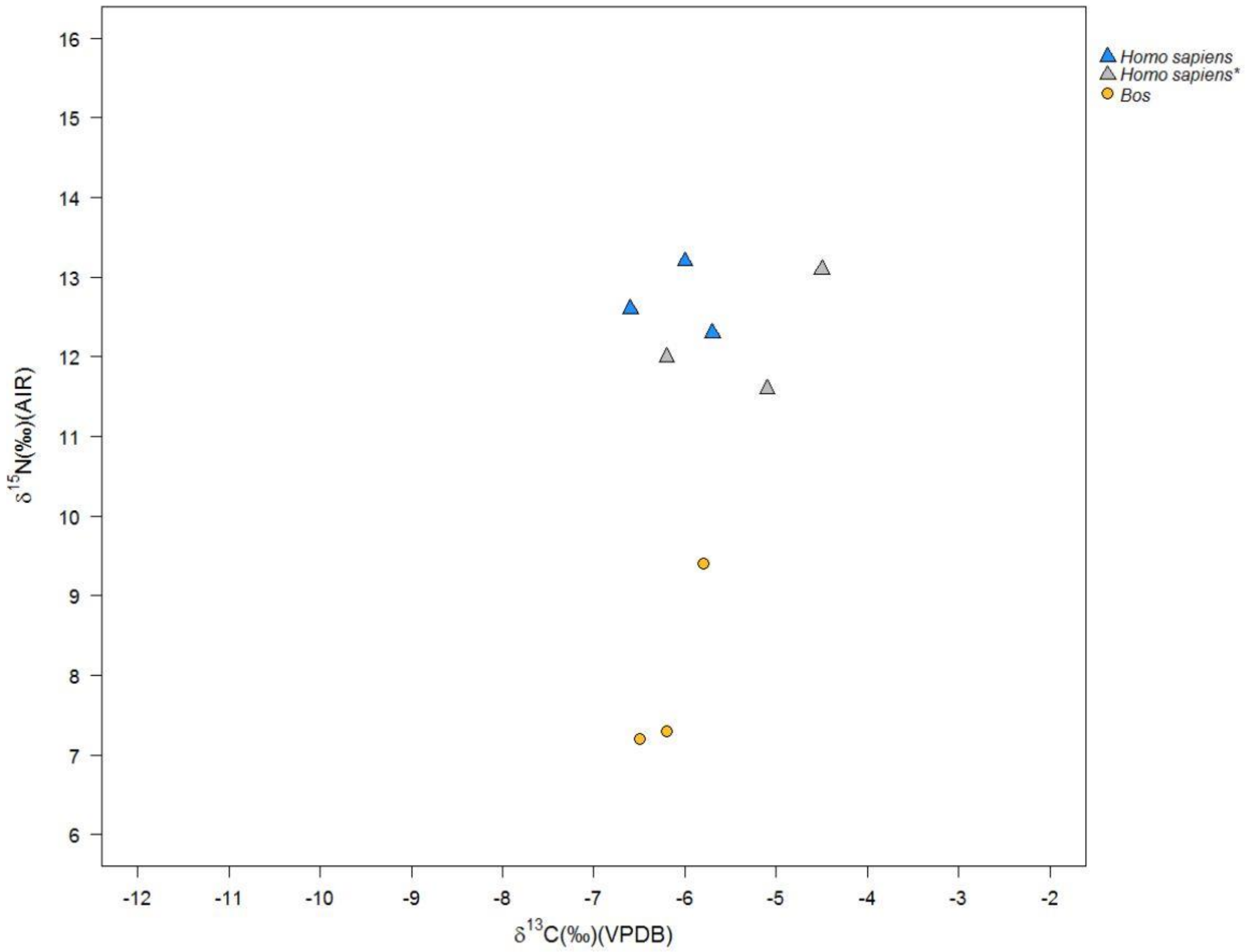

Supplementary Figure 10: $\delta^{15}N$ and $\delta^{13}C$ measurements for human and faunal bone collagen for Lukenya Hill (GvJm202 and GvJm184). *results previously published in[14]

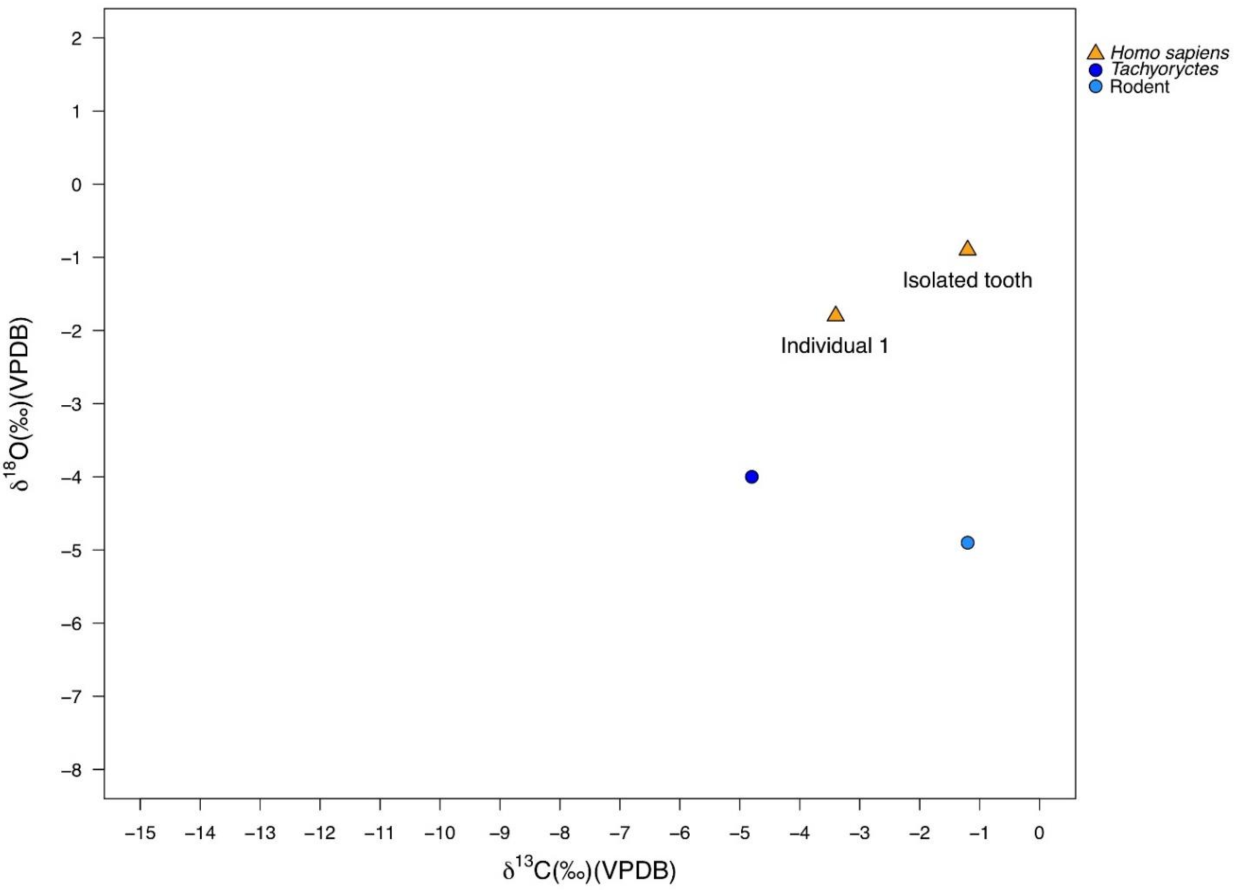

Supplementary Figure 11: $\delta^{13}C$ and $\delta^{18}O$ measurements for tooth enamel samples from humans and rodents from Cole's Burial.

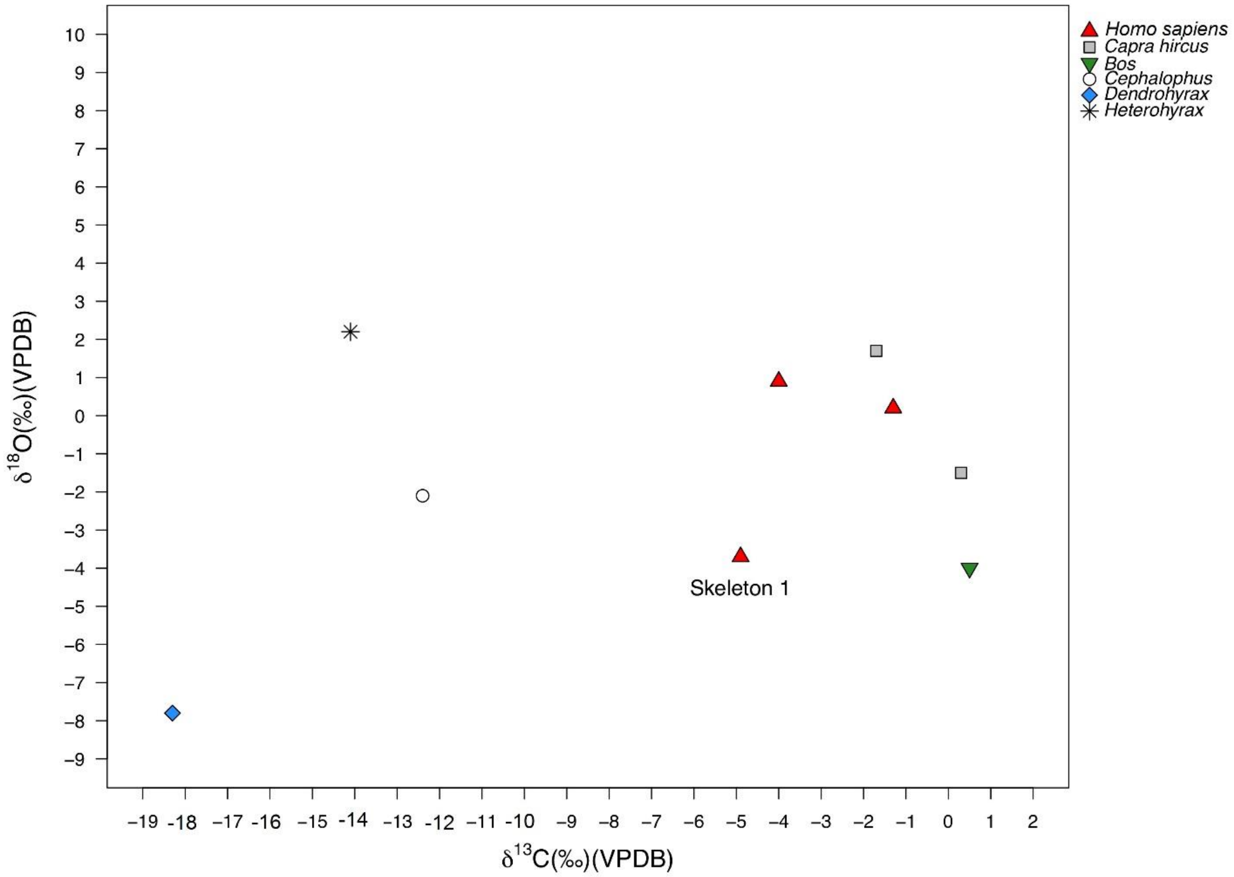

Supplementary Figure 12: $\delta^{13}C$ and $\delta^{18}O$ measurements for tooth enamel samples from humans and fauna from Molo Cave. Skeleton 1 was previously analysed for aDNA (MOL001)[13] and also had milk proteins in their dental calculus.

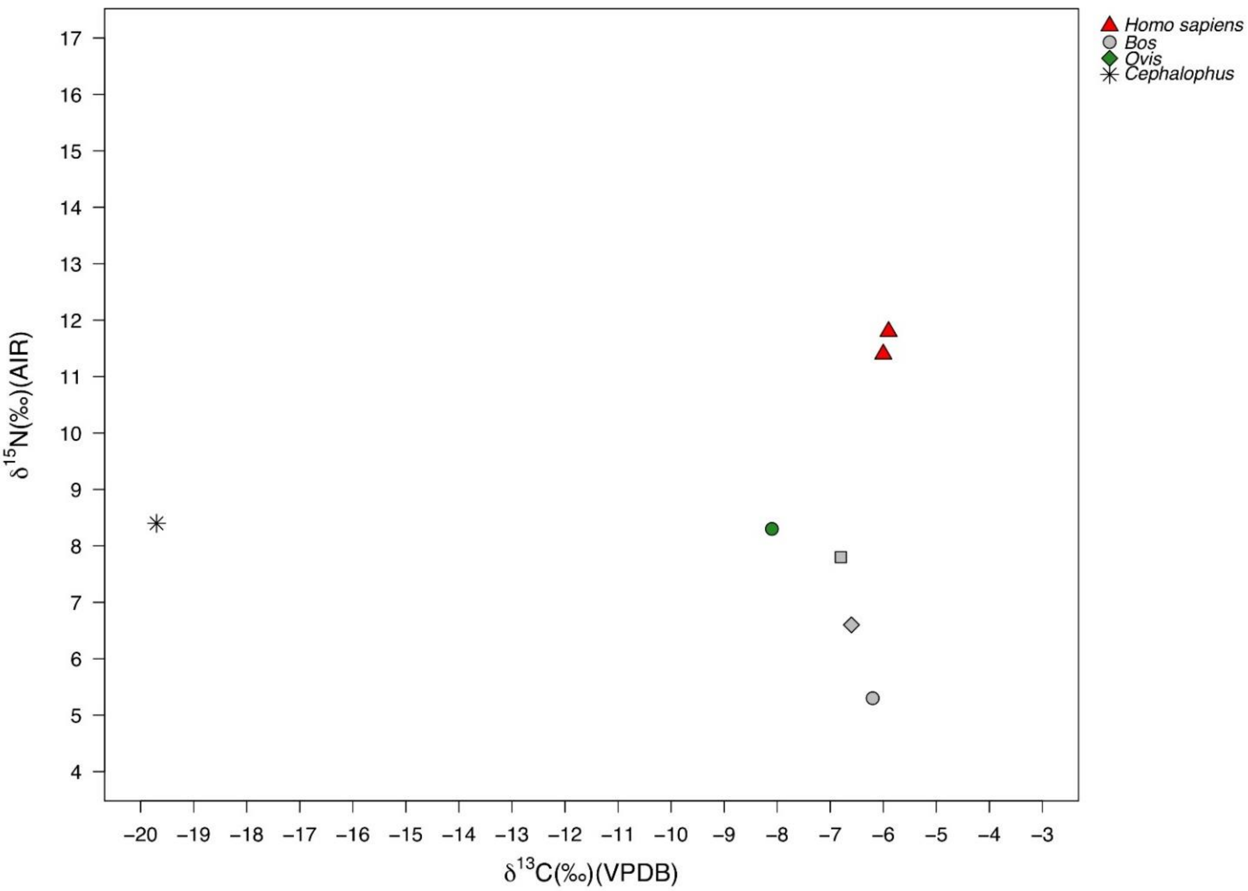

Supplementary Figure 13: $\delta^{15}N$ and $\delta^{13}C$ measurements for human and faunal bone collagen for Molo Cave.

Sample name	GrM	Calibrated dating result (95.4% probability)
DA-KAD10317-028.C	17738	2189 – 1978 calBC

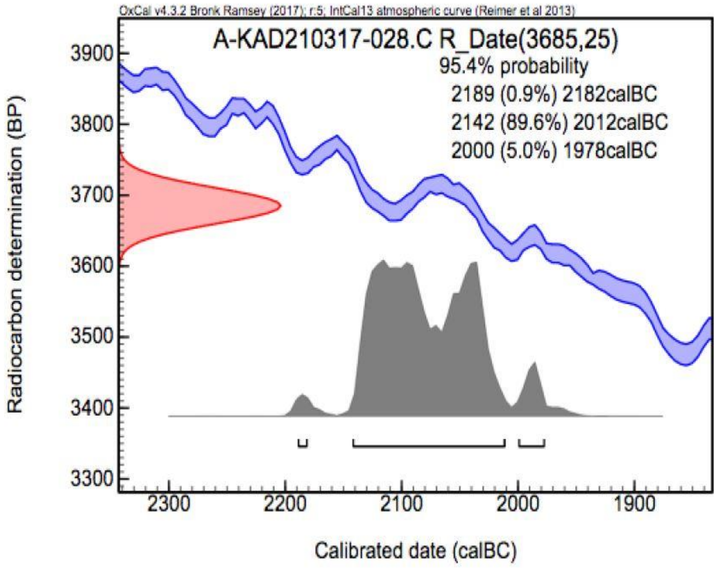

Supplementary Figure 14: Radiocarbon date for Kadruka 1 SK68. ^{14}C ages were calibrated to calendar years with software program: OxCal, version 4.3[68], using calibration curve: IntCal13[69].

Supplementary Table 1: Summary of all dental calculus samples studied, number that met OSSD criteria. *Radiocarbon dating of these remains was unsuccessful due to insufficient collagen.

Site	Country	Archaeological Period	Individuals/samples analysed	Individuals/samples passed OSSD	Individuals/samples with milk
Kadruka 1	Sudan	Neolithic-Kerma	5/10	3/4	1/1
Kadruka 21	Sudan	Neolithic	5/10	3/4	1/2
Berber Meroitic Cemetery	Sudan	Meroitic	5/5	1/1	1/1
Atbara Setiet West Bank	Sudan	Unknown*	2/2	1/1	0
Roseires East Bank	Sudan	Unknown*	1/1	0	0
Tinga Archaeological Rescue Project	Sudan	Napata -Meroitic	3/3	1/1	0
Kweka cemetery	Sudan	Unknown	2/2	0	0
Kadakol Christian Cemetery	Sudan	Christian Period	1/1	0	0
Lukenya Hill (GvJm202)	Kenya	Pastoral Neolithic	5/5	4/4	2/2
Molo Cave (GoJi3)	Kenya	Pastoral Neolithic	2/2	2/2	1/1
Cole’s Burial (GrJj5a)	Kenya	Pastoral Neolithic	3/3	3/3	2/2
Pickford's Site (GvJn14)	Kenya	Pastoral Neolithic	1/1	0	0
Jarigole (GbJ1)	Kenya	Pastoral Neolithic	3/3	0	0
Njoro River Cave (GrJh4)	Kenya	Pastoral Neolithic/Elmenteitan	3/3	1/1	0
**Total (individuals/samples)**			**41/51**	**19/21**	**8/9**

Supplementary Table 2: Summary of individuals with dairy proteins and radiocarbon dates. Lab codes are only reported for individuals with direct dates as opposed to associated dates from the same site. Dates published in *[13], †[16], ‡Associated date for all burials at GvJm202.

Site	Context	Archaeological Period	Dates cal. BP (Lab code)	Genetic cluster	LP alleles?	Species associated with milk proteins
Kadruka 1	KDK1 SK68	Neolithic-Kerma	4139-3928 (GrM 17738)	Analysis failed	n/a	*Capra*, Caprinae, Bovidae, Pecora
Kadruka 21	KDK21 SK129	Neolithic	Analysis failed	Analysis failed	n/a	Bovinae/*Ovis*, Bovidae
Berber Meroitic Cemetery	BMC 2015 T38 B	Meroitic	n/a	n/a	n/a	Pecora, Bovinae/*Ovis*, Bovinae, Bos, Bovidae
Lukenya Hill (GvJm202)	Skeleton A 533	Pastoral Neolithic	3610–3460*‡	n/a	n/a	Pecora, Bovinae/*Ovis*
Lukenya Hill (GvJm202)	703; West rocky sect; DD 75.5-85.5	Pastoral Neolithic	3610–3460*‡	n/a	n/a	Bovidae, Bovinae/*Ovis*
Molo Cave (GoJi3)	Skeleton 1, 55	Pastoral Neolithic	1415–1320 (OxA-37, 359)*	East Africa Pastoral*	No	Bovinae/*Ovis*
Cole's Burial (GrJj5a)	Individual 1, 107	Pastoral Neolithic	3351-3180 (PSU I8874)†	Pastoral Neolithic**	No	Pecora, Bovidae, Bovinae/*Ovis*
Cole's Burial (GrJj5a)	21, 5a, isolated tooth	Pastoral Neolithic	n/a	n/a	n/a	Pecora, Bovidae, Bovinae/*Ovis*

Supplementary Table 3: Number of possible deamidation sites for milk proteins identified in this study per individual. For two samples (DA356 and DA324) milk proteins were identified but they did not meet the criteria (see methods). These were therefore not reported as evidence of milk consumption in the main text.

**Site**	**Sample**	**Protein**	**Total per individual (without species-specific sites)**	**Deamidated**	**Site-specific (in all cases these are the deamidated versions)**
Kadruka 1	Z708	LACB	3	3	3
Kadruka 21	Z452	LACB	0	0	5
	DA351	CASB	2	0	0
Berber Meroitic Cemetery	DA156	LACB, CASA2, CASB, CASK	17	1	5
Lukenya Hill	DA145	LACB	0	0	3
	DA323	LACB	1	0	4
	DA356	*LACB*	*0*	*0*	*3*
Cole's Burial	DA325	LACB	4	3	3
	DA346	LACB	2	0	5
	DA324	*LACB*	*0*	*0*	*2*
Molo Cave	DA144	LACB	0	0	7

Supplementary Table 4: Summary of species information for peptide sequences. Pass for BLAST is 100% homology and 100% coverage to only the desired protein.

Protein	Start Position	Sequence	BLAST	Geneious
CASA2	130	NAVPITPTLNR	Pass	Bovinae
CASA2	153	TVDMESTEVFTK	Pass	Bovinae
CASA2	48	ENLCSTFCK	Pass	Bovinae
CASB	192	AVPYPQR	Pass	Bovinae
CASB	185	VLPVPQK	Pass	Bovidae
CASB	208	YQEPVLGPVRGPF	Pass	Bovidae
CASK	46	YIPIQYVLSR	Pass	Bovidae
CASK	108	SCQAQPTTMAR	Pass	Bovinae
CASK	90	SPAQILQWQVLSNTVPAK	Pass	*Bos*
CASK	173	VIESPPEIN	Pass	Bovinae
LACB	57	VYVEELKPTPEGDLEILLQK	Pass	Bovidae
LACB	92	TKIPAVFK	Pass	Bovidae
LACB	94	TKIPAVFKIDAL	Pass	Bovidae
LACB	94	TKIPAVFKID	Pass	Bovidae
LACB	96	IPAVFKID	Pass	Pecora
LACB	100	IDALNENK	Pass	Pecora
LACB	100	IDALNENKVLVLDTDYKK	Pass	Pecora
LACB	108	VLVLDTDYKK	Pass	Pecora
LACB	108	VLVLDTDYK	Pass	Pecora
LACB	110	VLVL	Fail	Fail
LACB	134	SLACQCLVR	Pass	Pecora
LACB	141	TPEVDDEALEKFDKALK	Pass	Bovinae or *Ovis*
LACB	141	TPEVDDEALEKFDK	Pass	Bovinae or *Ovis*
LACB	141	TPEVDDEALEK	Pass	Bovinae or *Ovis*
LACB	143	TPEVDKEALEKFDK	Pass	*Capra*
LACB	143	TPEVDKEALEK	Pass	*Capra*
LACB	149	EALEKFDK	Fail	Fail
LACB	167	LAFNPTQLEGQCHV	Pass	Caprinae
LACB	167	LAF	Fail	Fail

Supplementary Table 5: Taxonomic information for species for casein proteins and Beta-lactoglobulin.

**Order**	**Suborder**	**Infraorder**	**Family**	**Subfamily**	**Genus**	**Species**	**CASA2**	**CASB**	**CASK**	**LACB**
Cetartiodactyla	Ruminantia	Pecora	Bovidae	Bovinae	*Bos*	*mutus*	x	x	x	x
Cetartiodactyla	Ruminantia	Pecora	Bovidae	Bovinae	*Bos*	*taurus*	x	x	x	x
Cetartiodactyla	Ruminantia	Pecora	Bovidae	Bovinae	*Bos*	*indicus*		x	x	
Cetartiodactyla	Ruminantia	Pecora	Bovidae	Bovinae	*Bubalus*	*bubalis*	x	x	x	x
Cetartiodactyla	Ruminantia	Pecora	Bovidae	Caprinae	*Ovis*	*aries*	x	x	x	x
Cetartiodactyla	Ruminantia	Pecora	Bovidae	Caprinae	*Ovis*	*orientalis*			x	
Cetartiodactyla	Ruminantia	Pecora	Bovidae	Caprinae	*Ovis*	*vignei*			x	
Cetartiodactyla	Ruminantia	Pecora	Bovidae	Caprinae	*Capra*	*hircus*	x	x	x	x
Cetartiodactyla	Ruminantia	Pecora	Bovidae	Caprinae	*Pantholops*	*hodgsonii*	x			
Cetartiodactyla	Ruminantia	Pecora	Bovidae	Caprinae	*Naemorhedus*	*goral*			x	
Cetartiodactyla	Ruminantia	Pecora	Bovidae	Caprinae	*Oreamnos*	*americanus*			x	
Cetartiodactyla	Ruminantia	Pecora	Cervidae		*Rangifer*	*tarandus*				x
Cetartiodactyla	Tylopoda		Camelidae		*Camelus*	*bactrianus*	x	x	x	
Cetartiodactyla		Suina	Suidae		*Sus*	*scrofa*	x	x	x	x
Cetartiodactyla	Odontoceti		Delphinidae		*Tursiops*	*truncatus*		x	x	x
Perissodactyla			Equidae		*Equus*	*asinus*	x	x		x
Perissodactyla			Equidae		*Equus*	*caballus*	x	x	x	x
Primates			Hominidae		*Homo*	*sapiens*		x	x	
Carnvora			Canidae		*Canis*	*lupus*				x
Carnvora			Felidae		*Felis*	*catus*				x

Supplementary Table 6: Identification of faunal remains for isotope analysis using morphometrics and ZooMS.

Type	Site	MPI-SHH Database	Lab ID	Element	Morphological ID	ZooMS ID	Final ID
Bone	Lukenya Hill (GvJm 184)	DA-LUKI0317-002	LUKF001	Humerus CYL	Bovid 3	*Bos*	*Bos*
	Lukenya Hill (GvJm 202)	DA-LUK0317-012	LUKF003	Long bone fr	Mammal ≥3	*Bos*	*Bos*
	Lukenya Hill (GvJm 202)	DA-LUK0317-014	LUKF004	Femur shfr.	Bovid 3	*Bos*	*Bos*
	Molo Cave (GoJi3)	DA-MOL0317-017	MOLF003	Left mandible	Bovid 1 (neonate)	*Bos*/Bison/*Cephalophus*	*Cephalophus* sp.
	Molo Cave (GoJi3)	DA-MOL0317-004.B	MOLF005	R rib angle fr	Bovid 3	*Bos*	*Bos*
	Molo Cave (GoJi3)	DA-MOL0317-007	MOLF008	Non ID fragment	Mammal ≥3	*Bos*	*Bos*
	Molo Cave (GoJi3)	DA-MOL0317-023	MOLF010	Rib proximal fr	Bovid 2	*Ovis*	*Ovis*
	Molo Cave (GoJi3)	DA-MOL0317-008a	MOLF011	Upper limb bone fragment	Mammal ≥3	*Bos*	*Bos*
Tooth	Lukenya Hill (GvJm 202)	DA-LUK0317-013	LUKF002	LUM1	*cf. Eudorcas thomosonii*	n/a	*Eudorcas thomosonii*
	Cole's Burial	DA-COL0317-033	COLF002	RLI1	Rodent indet	n/a	Rodent
	Cole's Burial	DA-COL0317-004	COLF003	LLI1	*Tachyoryctes*	n/a	*Tachyoryctes*
	Molo Cave (GoJi3)	DA-MOL0317-011	MOLF001	LLM3	*Capra hircus*	n/a	*Capra hircus*
	Molo Cave (GoJi3)	DA-MOL0317-009	MOLF002	LUP0	*cf. Bos*	n/a	*Bos*
	Molo Cave (GoJi3)	DA-MOL0317-017	MOLF003	LLdp4	Bovid 1 (neonate)	n/a	*Cephalophus* sp.
	Molo Cave (GoJi3)	DA-MOL0317-004.A	MOLF004	LLM2	*Capra hircus*	n/a	*Capra hircus*
	Molo Cave (GoJi3)	DA-MOL0317-010.A	MOLF006	RUP2	*Dendrohyrax*	n/a	*Dendrohyrax*
	Molo Cave (GoJi3)	DA-MOL0317-022	MOLF007	RLP4	*Heterohyrax*	n/a	*Heterohyrax*

Supplementary Information References

1. Reinold. *Kadruka and the Neolithic in the Northern Dongola Reach*. (2001).
2. Reinold, J. Kadruka. in *Sudan: ancient treasures: an exhibition of recent discoveries from the Sudan National Museum* (eds. Welsby, D. A. & Anderson, J. R.) 42–48 (British Museum Press, 2004).
3. Ryder, M. L. Sheepskin from ancient Kerma, norther Sudan. *Oxford J Archeol* **6**, 369–380 (1987).
4. Chaix, L. Rapport preliminaire sur la faune du site de Kadruka I, Soudan Nord (Neolithique et Protohistorique). in *Archäologie du Nil Moyen, Vol. 2* (ed. Geus, F.) 61–62 (1987).
5. Bogucki, P. I. Ceramic Sieves of the Linear Pottery Culture and their economic implications. *Oxford Journal of Archaeology* **3**, 15–30 (1984).
6. Salque, M. *et al.* Earliest evidence for cheese making in the sixth millennium BC in northern Europe. *Nature* **493**, 522–535 (2012).
7. Bashir, M. S. & David, R. The Meroitic Cemetery at Berber. Recent Fieldwork and Discussion on Internal Chronology. *Sudan and Nubia, Bulletin No.19, The Sudan Archaeological Research Society* 97–105 (2015).
8. Tryon, C. A. *et al.* Late Pleistocene age and archaeological context for the hominin calvaria from GvJm-22 (Lukenya Hill, Kenya). *Proc. Natl. Acad. Sci. U. S. A.* **112**, 2682–2687 (2015).
9. Marshall, F. *et al.* Ancient herders enriched and restructured African grasslands. *Nature* **561**, 387–390 (2018).
10. Nelson, C. M. & Kimegich, J. Early development of pastoral adaptation in the central highlands of Kenya. in *Origin and Early Development of Food – Producing Cultures in North-Eastern Africa* (ed. Kryzaniak, L.) 481–487 (Poznan Archaeological Museum, 1984).
11. Sawchuk, E. A. Social change and human population movements — dental morphology in Holocene Eastern Africa. (University of Toronto, 2017). doi:10.1080/0067270X.2018.1525835.
12. Schepartz, L. A. From Hunters to Herders: Subsistence Pattern and Morphological Change in Eastern Africa. (University of Michigan., 1987).
13. Wang, K. *et al.* Ancient genomes reveal complex patterns of population movement, interaction, and replacement in sub-Saharan Africa. *Science Advances*, ***6*** (2020)
14. Ambrose, S. H. & DeNiro, M. J. Reconstruction of African human diet using bone collagen carbon and nitrogen isotope ratios. *Nature* **319**, 321–324 (1986).
15. Ambrose, S. H. The Introduction of Pastoral Adaptations to the Highlands of East Africa. in *From Hunters to Farmers: The Causes and Consequences of Food Production in Africa,* (eds. Clark, C. D. & Brandt, S. A.) 212–239 (University of California Press, Berkeley, CA, 1984).
16. Prendergast, M. E. *et al.* Ancient DNA reveals a multistep spread of the first herders into sub-Saharan Africa. *Science* **365**, (2019).
17. Wiśniewski, J. R., Zougman, A., Nagaraj, N. & Mann, M. Universal sample preparation method for proteome analysis. *Nat. Methods* **6**, 359–362 (2009).
18. Jeong, C. *et al.* Bronze Age population dynamics and the rise of dairy pastoralism on the eastern Eurasian steppe. *Proc. Natl. Acad. Sci. U. S. A.* **115**, E11248–E11255 (2018).
19. Charlton, S. *et al.* New insights into Neolithic milk consumption through proteomic analysis of dental calculus. *Archaeol. Anthropol. Sci.* **11**, 6183–6196 (2019).
20. Wilkin, S. *et al.* Dairy pastoralism sustained eastern Eurasian steppe populations for 5,000 years. *Nature Ecology & Evolution* **4**, 346–355 (2020).
21. Warinner, C. *et al.* Direct evidence of milk consumption from ancient human dental calculus. *Sci. Rep.* **4**, 7104 (2014).

22. Sielaff, M. *et al.* Evaluation of FASP, SP3, and iST Protocols for Proteomic Sample Preparation in the Low Microgram Range. *J. Proteome Res.* **16**, 4060–4072 (2017).
23. Hughes, C. S. *et al.* Single-pot, solid-phase-enhanced sample preparation for proteomics experiments. *Nat. Protoc.* (2018) doi:10.1038/s41596-018-0082-x.
24. Cleland, T. P. Human Bone Paleoproteomics Utilizing the Single-Pot, Solid-Phase-Enhanced Sample Preparation Method to Maximize Detected Proteins and Reduce Humics. *J. Proteome Res.* **17**, 3976–3983 (2018).
25. Batth, T. S. *et al.* Protein Aggregation Capture on Microparticles Enables Multipurpose Proteomics Sample Preparation. *Mol. Cell. Proteomics* **18**, 1027–1035 (2019).
26. Hendy, J. *et al.* Proteomic evidence of dietary sources in ancient dental calculus. *Proc. Biol. Sci.* **285**, (2018).
27. van Doorn, N. L., Wilson, J., Hollund, H., Soressi, M. & Collins, M. J. Site-specific deamidation of glutamine: a new marker of bone collagen deterioration. *Rapid Commun. Mass Spectrom.* **26**, 2319–2327 (2012).
28. Simpson, J. P. *et al.* The effects of demineralisation and sampling point variability on the measurement of glutamine deamidation in type I collagen extracted from bone. *J. Archaeol. Sci.* **69**, 29–38 (2016).
29. Ramsøe, A. *et al.* DeamiDATE 1.0: Site-specific deamidation as a tool to assess authenticity of members of ancient proteomes. *J. Archaeol. Sci.* **115**, 105080 (2020).
30. Robinson, N. E. & Robinson, A. B. Prediction of protein deamidation rates from primary and three-dimensional structure. *Proc. Natl. Acad. Sci. U. S. A.* **98**, 4367–4372 (2001).
31. Jersie-Christensen, R. R. *et al.* Quantitative metaproteomics of medieval dental calculus reveals individual oral health status. *Nat. Commun.* **9**, 4744 (2018).
32. Velsko, I. M. *et al.* Microbial differences between dental plaque and historic dental calculus are related to oral biofilm maturation stage. *Microbiome* **7**, 102 (2019).
33. Tanner, A. C. R. & Izard, J. Tannerella forsythia, a periodontal pathogen entering the genomic era. *Periodontol. 2000* **42**, 88–113 (2006).
34. Warinner, C. *et al.* Pathogens and host immunity in the ancient human oral cavity. *Nat. Genet.* **46**, 336–344 (2014).
35. Chen, T. *et al.* The Human Oral Microbiome Database: a web accessible resource for investigating oral microbe taxonomic and genomic information. *Database* **2010**, baq013 (2010).
36. Tsutaya, T. *et al.* Palaeoproteomic identification of breast milk protein residues from the archaeological skeletal remains of a neonatal dog. *Sci. Rep.* **9**, 12841 (2019).
37. Richter, K. K. *et al.* What's the Catch?: Archaeological application of rapid collagen-based species identification for Pacific Salmon. *J. Archaeol. Sci.***116**, (2020).
38. Bern, M., Kil, Y. J. & Becker, C. Byonic: advanced peptide and protein identification software. *Curr. Protoc. Bioinformatics* **Chapter 13**, Unit13.20 (2012).
39. Desiere, F. *et al.* The PeptideAtlas project. *Nucleic Acids Res.* **34**, D655–8 (2006).
40. Demarchi, B. *et al.* Protein sequences bound to mineral surfaces persist into deep time. *Elife* **5**, (2016).
41. Hendy, J. *et al.* A guide to ancient protein studies. *Nat Ecol Evol* (2018) doi:10.1038/s41559-018-0510-x.
42. Ambrose, S. H. Preparation and characterization of bone and tooth collagen for isotopic analysis. *J. Archaeol. Sci.* **17**, 431–451 (1990).
43. Calvin, M. & Benson, A. A. The path of carbon in photosynthesis. *Science* **107**, 476–480 (1948).
44. Hatch, M. D. & Slack, C. R. Photosynthesis by sugar-cane leaves. A new carboxylation reaction and the pathway of sugar formation. *Biochem. J* **101**, 103–111 (1966).
45. O'Leary, M. H. Carbon isotope fractionation in plants. *Phytochemistry* **20**, 553–567 (1981).
46. DeNiro, M. J. & Epstein, S. Influence of diet on the distribution of carbon isotopes in animals. *Geochim. Cosmochim. Acta* **42**, 495–506 (1978).

47. Lee-Thorp, J. A., Sealy, J. C. & van der Merwe, N. J. Stable carbon isotope ratio differences between bone collagen and bone apatite, and their relationship to diet. *J. Archaeol. Sci.* **16**, 585–599 (1989).
48. Bocherens, H. & Drucker, D. Trophic level isotopic enrichment of carbon and nitrogen in bone collagen: case studies from recent and ancient terrestrial ecosystems. *Int. J. Osteoarchaeol.* **13**, 46–53 (2003).
49. Schoeninger, M. J. & DeNiro, M. J. Nitrogen and carbon isotopic composition of bone collagen from marine and terrestrial animals. *Geochim. Cosmochim. Acta* **48**, 625–639 (1984).
50. Guiry, E. Complexities of Stable Carbon and Nitrogen Isotope Biogeochemistry in Ancient Freshwater Ecosystems: Implications for the Study of Past Subsistence and Environmental Change. *Frontiers in Ecology and Evolution* **7**, 313 (2019).
51. Ambrose, S. H. & Norr, L. Experimental Evidence for the Relationship of the Carbon Isotope Ratios of Whole Diet and Dietary Protein to Those of Bone Collagen and Carbonate. in *Prehistoric Human Bone: Archaeology at the Molecular Level* (eds. Lambert, J. B. & Grupe, G.) 1–37 (Springer Berlin Heidelberg, 1993).
52. Dansgaard, W. Stable isotopes in precipitation. *Tell'Us* **16**, 436–468 (1964).
53. Buchmann, N. & Ehleringer, J. R. CO2 concentration profiles, and carbon and oxygen isotopes in C3 and C4 crop canopies. *Agric. For. Meteorol.* **89**, 45–58 (1998).
54. Pellegrini, M., Pouncett, J., Jay, M., Pearson, M. P. & Richards, M. P. Tooth enamel oxygen 'isoscapes' show a high degree of human mobility in prehistoric Britain. *Sci. Rep.* **6**, 34986 (2016).
55. di Lernia, S. *et al.* Inside the 'African cattle complex': animal burials in the holocene central Sahara. *PLoS One* **8**, e56879 (2013).
56. Balasse, M., Ambrose, S. H., Smith, A. B. & Price, T. D. The Seasonal Mobility Model for Prehistoric Herders in the South-western Cape of South Africa Assessed by Isotopic Analysis of Sheep Tooth Enamel. *J. Archaeol. Sci.* **29**, 917–932 (2002).
57. Balasse, M., Smith, A. B., Ambrose, S. H. & Leigh, S. R. Determining sheep birth seasonality by analysis of tooth enamel oxygen isotope ratios: The late stone age site of Kasteelberg (South Africa). *J. Archaeol. Sci.* **30**, 205–215 (2003).
58. Janzen, A., Balasse, M. & Ambrose, S. H. Early Pastoral Mobility and Seasonality in Kenya Assessed through Stable Isotope Analysis. *J. Archaeol. Sci.* 117 **(2020)**
59. Chritz, K. L. *et al.* Climate, ecology, and the spread of herding in eastern Africa. *Quat. Sci. Rev.* **204**, 119–132 (2019).
60. Sealy, J. Isotopic Evidence for the Antiquity of Cattle-Based Pastoralism in Southernmost Africa. *Journal of African Archaeology* **8**, 65–81 (2010).
61. Ambrose, S. H. Stable carbon and nitrogen isotope analysis of human and animal diet in Africa. *J. Hum. Evol.* **15**, 707–731 (1987).
62. Balasse, M. & Ambrose, S. H. Distinguishing sheep and goats using dental morphology and stable carbon isotopes in C 4 grassland environments. *J. Archaeol. Sci.* **32**, 691–702 (2005).
63. Ambrose, S. H. & DeNiro, M. J. The Isotopic Ecology of East African Mammals. *Oecologia* **69**, 395–406 (1986).
64. Prendergast, M. E., Janzen, A., Buckley, M. & Grillo, K. M. Sorting the sheep from the goats in the Pastoral Neolithic: morphological and biomolecular approaches at Luxmanda, Tanzania. *Archaeol. Anthropol. Sci.* **11**, 3047–3062 (2019).
65. Tieszen, L. L. & Fagre, T. Effect of diet quality and composition on the isotopic composition of respiratory CO2, bone collagen, bioapatite, and soft tissues. in *Prehistoric human bone—archaeology at the molecular level* (eds. Lambert, J. B. & Grupe, G.) 121–155 (Springer-Verlag, Berlin, 1993).
66. DeNiro, M. J. Postmortem preservation and alteration of in vivo bone collagen isotope ratios in relation to palaeodietary reconstruction. *Nature* **317**, 806 (1985).
67. van Klinken, G. J. Bone Collagen Quality Indicators for Palaeodietary and Radiocarbon Measurements. *J. Archaeol. Sci.* **26**, 687–695 (1999).

68. Bronk Ramsey, C. Methods for Summarizing Radiocarbon Datasets. *Radiocarbon* **59**, 1809–1833 (2017).
69. Reimer, P. J. *et al.* IntCal13 and Marine13 Radiocarbon Age Calibration Curves 0–50,000 Years cal BP. *Radiocarbon* **55**, 1869–1887 (2013).
