# Supplementary material for: Ancient proteins provide evidence of dairy consumption in eastern Africa
Source: Nat Commun. 2021 Jan 27;12:632. doi: 10.1038/s41467-020-20682-3 (PMC7841170; doi:10.1038/s41467-020-20682-3)
Supplement: Supplementary file 3 — Description of Additional Supplementary Files [file 41467_2020_20682_MOESM3_ESM.pdf]

Description of Additional Supplementary Files

File Name: Supplementary Data 1
Description: Summary of all individuals from which calculus was sampled for proteomic analysis.

File Name: Supplementary Data 2
Description: Total proteins identified in all samples. All samples were searched using both MASCOT and Byonic.

File Name: Supplementary Data 3
Description: Results of searching against custom-made oral signature screening database (OSSD). OSSD pass is at ten or more total proteins and at least 45% match (oral+immune).

File Name: Supplementary Data 4
Description: Mascot error-tolerant search results of samples that passed OSSD screening filtered for counts of amino acids >4.

File Name: Supplementary Data 5
Description: Byonic error-tolerant search results for samples that passed OSSD screening.

File Name: Supplementary Data 6
Description: Summary of milk proteins identified in this study per individual. All samples were searched using both Mascot and Byonic. For two samples (DA356 and DA324) milk proteins were identified but they did not meet the criteria (see methods). These were therefore not reported as evidence of milk consumption in the main text.

File Name: Supplementary Data 7
Description: ZooMS results.

File Name: Supplementary Data 8
Description: $\delta^{15}N$ and $\delta^{13}C$ values for human and animal bulk bone collagen.

File Name: Supplementary Data 9
Description: $\delta^{13}C$ and $\delta^{18}O$ values for human and animal tooth enamel.
