# Supplementary material for: Ancient proteins provide evidence of dairy consumption in eastern Africa
Source: Nat Commun. 2021 Jan 27;12:632. doi: 10.1038/s41467-020-20682-3 (PMC7841170; doi:10.1038/s41467-020-20682-3)
Supplement: Supplementary file 5 — Reporting Summary [file 41467_2020_20682_MOESM5_ESM.pdf]

nature research

Corresponding author(s): Madeleine Bleasdale, Nicole Boivin

Last updated by author(s): 2020/12/08

Reporting Summary

Nature Research wishes to improve the reproducibility of the work that we publish. This form provides structure for consistency and transparency in reporting. For further information on Nature Research policies, see Authors & Referees and the Editorial Policy Checklist .

Statistics

For all statistical analyses, confirm that the following items are present in the figure legend, table legend, main text, or Methods section.

n/a	Confirmed	
☐	☒	The exact sample size (n) for each experimental group/condition, given as a discrete number and unit of measurement
☐	☒	A statement on whether measurements were taken from distinct samples or whether the same sample was measured repeatedly
☒	☐	The statistical test(s) used AND whether they are one- or two-sided<br>*Only common tests should be described solely by name; describe more complex techniques in the Methods section.*
☒	☐	A description of all covariates tested
☒	☐	A description of any assumptions or corrections, such as tests of normality and adjustment for multiple comparisons
☒	☐	A full description of the statistical parameters including central tendency (e.g. means) or other basic estimates (e.g. regression coefficient) AND variation (e.g. standard deviation) or associated estimates of uncertainty (e.g. confidence intervals)
☒	☐	For null hypothesis testing, the test statistic (e.g. F, t, r) with confidence intervals, effect sizes, degrees of freedom and P value noted<br>*Give P values as exact values whenever suitable.*
☒	☐	For Bayesian analysis, information on the choice of priors and Markov chain Monte Carlo settings
☒	☐	For hierarchical and complex designs, identification of the appropriate level for tests and full reporting of outcomes
☒	☐	Estimates of effect sizes (e.g. Cohen's d, Pearson's r), indicating how they were calculated

Our web collection on statistics for biologists contains articles on many of the points above.

Software and code

Policy information about availability of computer code

Data collection	No software was used for data collection
Data analysis	Tandem mass spectra were extracted by MSConvert version 3.0.11781. Alignment search of all milk peptide spectral matches was performed using NCBI BLAST web interface.

For manuscripts utilizing custom algorithms or software that are central to the research but not yet described in published literature, software must be made available to editors/reviewers. We strongly encourage code deposition in a community repository (e.g. GitHub). See the Nature Research guidelines for submitting code & software for further information.

Data

Policy information about availability of data

All manuscripts must include a data availability statement. This statement should provide the following information, where applicable:

- Accession codes, unique identifiers, or web links for publicly available datasets
- A list of figures that have associated raw data
- A description of any restrictions on data availability

Raw and processed MS/MS files are available to download via MassIVE repository with accession code MSV000085058 [doi:10.25345/C55M4S]. The full Oral Signature Screening Database (OSSD) and associated results are available via MassIVE with accession code MSV000086557 [doi:10.25345/C5PR4T] and on the open-access repository Zenodo [https://doi.org/10.5281/zenodo.3698271]. The SP3 protocol is published open-access on protocols.io [dx.doi.org/10.17504/protocols.io.bfgrjjv6]. All other supporting data are available within the paper and supplementary information files.

Field-specific reporting

Please select the one below that is the best fit for your research. If you are not sure, read the appropriate sections before making your selection.

☒ Life sciences ☐ Behavioural & social sciences ☐ Ecological, evolutionary & environmental sciences

For a reference copy of the document with all sections, see nature.com/documents/nr-reporting-summary-flat.pdf

Life sciences study design

All studies must disclose on these points even when the disclosure is negative.

Sample size	The number of individuals analysed was selected based on the presence of ancient dental calculus deposits and preservation of archaeological bones and teeth. Dental calculus was analysed from humans (n=51, representing 41 individuals) previously excavated from 13 archaeological sites spread across Kenya and Sudan. In addition, archaeological bones (n=13) and teeth (n=21) were sampled from a subset of human individuals for stable carbon (δ13C), nitrogen (δ15N) and oxygen (δ18O) isotope analysis. Enamel and bones were also sampled from associated fauna (including Bos, Cephalophus sp., Ovis, Eudorcas thomosonii, Capra hircus and Dendrohyrax) to create a dietary isotopic baseline. For highly fragmented animal bones (n=8) collagen left over from isotope pretreatment was analysed by Zooarchaeology by Mass Spectrometry (ZooMS) to confirm taxonomic identifications. Five samples (1 bone fragment from petrous portion, 3 teeth and 1 hair sample) from human burials were sent for radiocarbon dating and were split between the Centre for Isotope Research Groningen (CIO, Lab ID: GrM) and Scottish Universities Environmental Research Centre Radiocarbon Laboratory Glasgow (SUERC, Lab ID: GU). Only the hair sample yielded a successful result.
Data exclusions	Some dental calculus samples did not have any oral signature after assessment against our Oral Signature Screening Database (OSSD) and were therefore excluded from final data analysis (error-tolerant searches using Mascot and Byonic).
Replication	A subset of 10 dental calculus samples were extracted with two different methods (SP3 and FASP). All other calculus samples were extracted with FASP. No replication was performed due to small quantities of starting material.<br><br>For stable isotope analysis, collagen was run in duplicate on the EA-IRMS. Long-term machine error was calculated based on replicate measurements over a year at ± 0.2‰ for δ13C and±0.2‰ for δ15N. Overall measurement precision was studied through the measurement of repeats of fish gelatin (n= 80, ± 0.2‰ for δ13C and±0.2‰ for δ15N). Machine error based on the analyses of standards is ± 0.1‰ for δ13C and±0.2‰ for δ18O. Overall measurement precision was assessed through repeat measurements of MERCK CaCO3 (n= 20, ± 0.2‰ for δ13C and±0.2‰ for δ18O, δ13C = ~ -40.6 ‰, δ18O= ~ -13.3 ‰) and an in-house equid tooth standard (n=10, ± 0.3‰ for δ13C and±0.2‰ for δ18O).
Randomization	Samples were run in a randomized order on the LC-MS/MS. A mixture of wild and domestic fauna were analysed for isotope analysis based on the preservation of skeletal elements.
Blinding	Blinding is not relevant as the experiments performed are based on all available material.

Reporting for specific materials, systems and methods

We require information from authors about some types of materials, experimental systems and methods used in many studies. Here, indicate whether each material, system or method listed is relevant to your study. If you are not sure if a list item applies to your research, read the appropriate section before selecting a response.

Materials & experimental systems

n/a	Involved in the study	
☒	☐	Antibodies
☒	☐	Eukaryotic cell lines
☐	☒	Palaeontology
☒	☐	Animals and other organisms
☒	☐	Human research participants
☒	☐	Clinical data

Methods

n/a	Involved in the study	
☒	☐	ChIP-seq
☒	☐	Flow cytometry
☒	☐	MRI-based neuroimaging

Palaeontology

Specimen provenance

Ancient dental calculus samples came from the following sites: Kadruka 1, Kadruka 21, Berber Meroitic Cemetery, Atbara West Bank, Roseires East Bank, Tinga Archaeological Rescue Project, Kweka cemetery, Katakol Christian Cemetery, Lukenya Hill (GvJm 202), Molo Cave (GoJi 3), Cole's Burial (GrJj 5a), Pickford's Site (GvJn 14), Jarigole (GbJ 1) and Njoro River Cave (GrJh 4). Human and faunal remains were collected during previous excavations and curated by the National Museums of Kenya, Nairobi; Laboratory of Prehistoric Archaeology and Anthropology, University of Geneva and the National Corporation for Antiquities and Museums of Sudan, Khartoum.

All material from Kenya was sampled and exported under permits issued by the National Museums of Kenya. Material from Kadruka 1 and Kadruka 21 was sampled and exported from the Laboratory of Prehistoric Archaeology and Anthropology, University of Geneva under the terms of an agreement with the Section française de la direction des antiquités au Soudan (SFDAS). All other archaeological remains from sites in Sudan were sampled and exported in accordance with section (31A) of the Sudan Antiquities Ordinance 1999. Permission "Ref. NCAM/4/B" was issued by the National Corporation for Antiquities and Museums (NCAM), Khartoum, Sudan.

Specimen deposition

Osteological remains are deposited at the Isotope Laboratory and Proteomic Laboratory of the Max Planck Institute for the Science of Human History, Jena, Germany.

Dating methods

A total of 5 samples were sent for radiocarbon dating and were split between the Centre for Isotope Research Groningen (CIO, Lab ID: GrM) and Scottish Universities Environmental Research Centre Radiocarbon Laboratory Glasgow (SUERC, Lab ID: GU). The hair sample from Kadruka 1 SK68 was pretreated with 4% HCl for a short period, rinsed with decarbonized water and dried before combustion. Four teeth were pretreated for dating at SUERC. A cranial fragment from individual Kadruka 21 Skeleton 129 was pretreated at CIO. Unfortunately none of the teeth or cranial bone fragment yielded sufficient collagen for dating. A radiocarbon date was successfully obtained from the hair sample from Kadruka 1 SK68. Radiocarbon ages were calibrated to calendar timescale using OxCal 4 and IntCal13 atmospheric calibration curve.

☒ Tick this box to confirm that the raw and calibrated dates are available in the paper or in Supplementary Information.
